# Oxalyl retro-peptide gelators. Synthesis, gelation properties and stereochemical effects

**DOI:** 10.3762/bjoc.6.106

**Published:** 2010-10-04

**Authors:** Janja Makarević, Milan Jokić, Leo Frkanec, Vesna Čaplar, Nataša Šijaković Vujičić, Mladen Žinić

**Affiliations:** 1 Laboratory for Supramolecular and Nucleoside Chemistry, Ruđer Bošković Institute, P.O.B. 180, HR-10002 Zagreb, Croatia

**Keywords:** chiral, organogel, oxalamide, retro-peptide, self-assembly

## Abstract

In this work we report on gelation properties, self-assembly motifs, chirality effects and morphological characteristics of gels formed by chiral retro-dipeptidic gelators in the form of terminal diacids (**1a–5a**) and their dimethyl ester (**1b**–**5b**) and dicarboxamide (**1c**–**5c**) derivatives. Terminal free acid retro-dipeptides (*S*,*S*)-bis(LeuLeu) **1a**, (*S,S*)-bis(PhgPhg) **3a** and (*S,S*)-bis(PhePhe) **5a** showed moderate to excellent gelation of highly polar water/DMSO and water/DMF solvent mixtures. Retro-peptides incorporating different amino acids (*S,S*)-(LeuPhg) **2a** and (*S,S*)-(PhgLeu) **4a** showed no or very weak gelation. Different gelation effectiveness was found for racemic and single enantiomer gelators. The heterochiral (*S,R*)**-1c** diastereoisomer is capable of immobilizing up to 10 and 4 times larger volumes of dichloromethane/DMSO and toluene/DMSO solvent mixtures compared to homochiral (*S,S*)**-1c.** Based on the results of ^1^H NMR, FTIR, CD investigations, molecular modeling and XRPD studies of diasteroisomeric diesters (*S,S*)**-1b/**(*S*,*R*)**-1b** and diacids (*S,S*)**-1b/**(*S*,*R*)**-1a**, a basic packing model in their gel aggregates is proposed. The intermolecular hydrogen bonding between extended gelator molecules utilizing both, the oxalamide and peptidic units and layered organization were identified as the most likely motifs appearing in the gel aggregates. Molecular modeling studies of (*S,S*)*-***1a/**(*S*,*R*)**-1a** and (*S,S*)**-1b/**(*S,R*)*-***1b** diasteroisomeric pairs revealed a decisive stereochemical influence yielding distinctly different low energy conformations: those of (*S*,*R*)-diastereoisomers with lipophilic *i*-Bu groups and polar carboxylic acid or ester groups located on the opposite sides of the oxalamide plane resembling bola amphiphilic structures and those of (*S,S*)-diasteroisomers possessing the same groups located at both sides of the oxalamide plane. Such conformational characteristics were found to strongly influence both, gelator effectiveness and morphological characteristics of gel aggregates.

## Introduction

Reversible processes of peptide, protein and nucleic acids self-assembly are of paramount importance in biotic systems and are central to vital biological functions. On the other hand, some pathological changes leading to diseases such as Alzheimer’s, Parkinson’s and prion diseases, type II diabetes, etc. are associated with anomalous self-assembly of smaller peptides into amyloid fibrils which finally result in the formation of amyloid plaques [1–4]. During the last two decades there has been a growing interest in self-organization of small peptide models capable of self-assembling into highly organized supramolecular structures with potential use as novel bio- or nano-materials possessing advanced properties and functions [5–10]. It has been shown that even short peptides, such as dipeptides, tripeptides or tetrapeptides, themselves or incorporated into more complex structures, are capable of self-assembling into fibers or fibrils [11–15]. In a number of cases such organization results in formation of gels consisting of self-assembled fibrous aggregates usually containing a large volume of solvent [16]. In gels, the fibers are heavily entangled into 3-dimensional networks which immobilize the solvent and prevent fluidity in the system [17–23]. In the last 15 years many low molecular weight gelling molecules of wide structural diversity, including a variety of amino acid and small peptide derivatives, have been prepared and studied [24–41]. These investigations revealed that gelator assemblies of various morphologies, including fibers and fiber bundles of diverse diameters, helical fibers or ribbons, tapes and nano-tubules, sometimes simultaneously present with micelles or vesicles, could be found [17–23]. For many gel systems evidence for hierarchical organisation was provided which determined the final morphological appearance of the aggregates [26]. It appears that gelation induced by aggregation of small abiotic or bio-inspired organic molecules represents an advantageous experimental system allowing in depth studies of the self-assembly as a general phenomenon. Such studies should ultimately result in revealing the relationship between gelator structures, self-assembly motifs and apparent morphologies of final assemblies as well as assisting in the elucidation of the role of solvent, which has been largely neglected in the majority of studies carried out to date. However, such an understanding of gelation is still out of reach; it is still hardly possible to predict gelation capability on the basis of the structure of a candidate molecule and it is even more difficult to predict which solvents and how effectively they would be gelled [42–43]. Hence, systematic studies of gels formed by structurally diverse small gelator molecules comprising elucidation of their self-assembly motifs, gelation effectiveness toward solvents of different structure and physical characteristics, estimation of solvation and stereochemical effects and their influence on the morphological characteristics of final gel assemblies may be rewarding, and should provide a much better understanding of the self-assembly processes involved in gelation.

In this work we report on gelation properties, self-assembly motifs, chirality effects and morphological characteristics of gels formed by chiral bis(dipeptide)oxalamides. Structurally, such gelators belong to the group of retro-peptides, which have been intensively studied as peptidomimetics due to their higher proteolytic stability and bioavailability compared to natural counterparts [44–47]. Despite very promising biomedicinal properties, very little is known about the self-assembly potential of this class of compounds in solution. Computer simulations of some malonamide retro-peptides have shown that the extended conformations are less stable than the helical ones [48–49]. Nevertheless, the crystal structure of the retro–inverso peptide Bz–S–gAla–R–mAla–NHPh revealed it’s unidirectional self-assembly by intermolecular β-sheet type of hydrogen bonding so that malonamide retro-peptides could be considered as potential candidates for development of new gelator molecules [50]. The oxalamide based retro-peptides are relatively rare and much less studied than the more flexible malonamide retro-peptides [51–54]. In contrast to the malonamide group, the planar and much more rigid oxalamide fragment is self-complementary and exhibit a strong tendency for intermolecular hydrogen bonding both in the solid state and in the solution [55–60]. Hence, the oxalyl retro-peptides are expected to preferably form extended conformations capable of intermolecular oxalamide–oxalamide hydrogen bonding and the formation of unidirectional assemblies the latter being a necessary condition for gelation [24–31,58–60]. Herein, we provide experimental and molecular modeling evidence that the oxalamide retro-peptides indeed tend to form unidirectional hydrogen bonded assemblies of gelator molecules that adopt fully extended conformations. We also present the evidence that for the (*S,R)*-bis(LeuLeu) **1a** and (*S,S)*-bis(LeuLeu) **1a** retro-peptidic gelators, the stereochemistry has a decisive impact on their gelation effectiveness and final gel morphology in their water/DMSO gels.

## Results and Discussion

### Synthesis of oxalyl retro-dipeptidic gelators

**Scheme 1:**
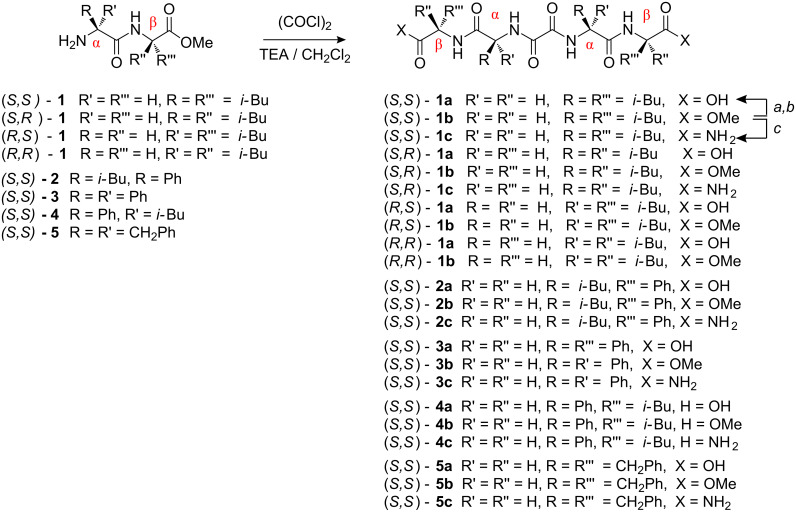
Oxalyl retro-dipetide gelators; each **b** to **a**, (*a*) LiOH/MeOH, H_2_O; (*b*) H^+^; each **b** to **c**: (*c*) NH_3_/MeOH.

A series of chiral bis(dipeptide)oxalamides was prepared as outlined in Scheme 1. The synthesis and analytical characterization of the prepared compounds are collected in the Supporting Information. Two sets of bis(dipeptide)oxalamides were prepared: the first incorporating a single amino acid and variable terminal groups such as carboxylic acid, methyl ester and carboxamide, namely (*S*,*S*)-bis(LeuLeu) **1a**, **b**, **c**; (*S,S*)-bis(PhgPhg) **3a**, **b**, **c** and (*S,S*)-bis(PhePhe) **5a**, **b**, **c** and, the second containing two different amino acids, (*S,S*)-bis(LeuPhg) **2a**, **b**, **c** and (*S,S*)-bis(PhgLeu) **4a**, **b**, **c** (configurations of only two of the four stereogenic centers are denoted corresponding to that of oxalamide α- and β-amino acid, respectively, as depicted in Scheme 1).

**Figure 1:**
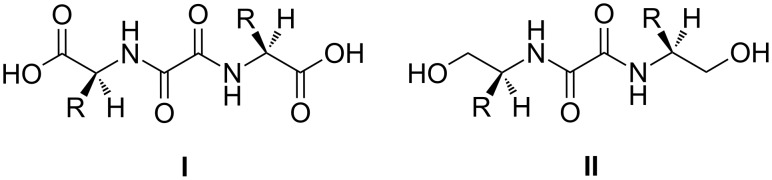
Chiral bis(amino acid)-(**I**) and bis(amino alcohol)-(**II**)-oxalamide gelators.

Compared to the previously studied bis(amino acid)-oxalamide gelators (Figure 1), the retro-dipeptidic gelators, in addition to the oxalamide hydrogen bonding unit, also contain two peptidic units with specifically oriented hydrogen bond donor and acceptor sites and amino acid lipophilic substituents. Such structural characteristics enable multiple structural and stereochemical variations of the basic gelator structure and subsequent studies of structural and stereochemical influences on gelation properties, self-assembly motifs and gel morphology.

The influence of stereochemistry on self-aggregation and morphology was studied with **1a–c** combining different configurations of Leu: (*S,R*)-**1a**, **b**, **c** and (*R,S*)-**1a**, **b**, **c**. Gelation properties of pure enantiomers (*S,S*)-**1b** and (*S,R*)-**1b** are compared with those of (*S,S*)-**1b**/(R,R)-**1b** and (*S,R*)-**1b**/(*R,S*)-**1b** racemic mixtures (Scheme 1).

### Gelation properties

#### Terminal diacid retro-dipeptides

Gelation observed for selected gelator–solvent pairs is expressed by gelator effectiveness (G_eff_, mL) corresponding to the maximal volume of solvent that could be immobilized by 10 mg of the gelator (Table 1). The oxalamides **1a**, **3a** and **5a** were insoluble in water but showed moderate to excellent gelation of water/DMSO and water/DMF solvent mixtures. The Leu containing gelator **1a** appeared more than 2 times more effective in gelation of water/DMSO or DMF mixtures than the aromatic acid containing gelators **3a** and **5a**. However, **3a** and **5a** also gelled small to moderate volumes of EtOH and *rac*-2-octanol, whilst Leu incorporating **1a** formed gels with the more lipophilic solvents, decalin and tetralin. The retro-dipeptides containing two different amino acids showed no or only weak gelation; (*S,S*)-(LeuPhg) **2a** lacked any gelation ability toward the tested solvents, while (*S,S*)-(PhgLeu) **4a** showed only weak gelation of water/DMSO, dichloromethane and toluene. Apparently, the retro-peptides incorporating aliphatic and aromatic amino acids are less versatile gelators compared to retro-peptides containing identical amino acid fragments. The latter points to the importance of intermolecular lipophilic interactions for the stabilization of gel assemblies being stronger in the case of identical amino acids either lipophilic or aromatic, and weaker for mixed aromatic-lipophilic amino acid fragments present in the gelator molecule.

Interestingly, the (*S,R*)-bis(LeuLeu) retro-peptide **1a**, the diastereomer of (*S,S*)-**1a**, exhibited an increased G_eff_ for the water/DMSO mixture and decalin which however is absent for water/DMF solvent mixture (Table 1). The latter exemplifies the strong stereochemical influence on gelator effectiveness in certain solvents.

**Table 1:** Gelator effectivenesses (G_eff_, mL) of retro-dipeptides **1a**–**5a** and bis(Leu)oxalamide **I** in gelation of various solvents and solvent mixtures (sol.: soluble; ins.: insoluble; cr.: crystallization; [A] gel/sol mixture).

Solvent	**I**	(*S,S*)**-1a**	(*S,R*)**-1a**	(*S,R*)-**1a**/ (*R,S*)-**1a**	(*S,S*)-**1a**/ (*R,R*)-**1a**	**2a**	**3a**	**4a**	**5a**

H_2_O	0.4	ins.	ins.	ins.	ins.	ins.	ins.	ins.	ins.
H_2_O/DMSO	0.8+0.4	5.09+2.98	15.0+4.5	5.3+5.3	cr.	cr.	2.3+1.6	1.0+1.0	1.1+0.5
H_2_O/DMF	cr.	5.84+3.25	7.5+2.0	2.8+1.7	cr.	cr.	2.5+1.1	cr.	2.8+0.7
EtOH	1.5	sol.	cr.	cr.	cr.	sol.	cr.	sol.	1.1
±2-octanol	10.95	ins.	sol.		cr.	sol.	1.25	cr.	5.75
THF	0.4	sol.	sol.	sol.	sol.	sol.	sol.	sol.	sol.
CH_2_Cl_2_	1.5+0.05^a^	ins.	cr.	ins.	ins.	ins.	ins.	0.25	ins.
CH_3_CN	0.95	cr.	cr.	cr.	cr.	sol.	cr.	ins.	7.0
toluene	1.95	cr.	cr.	0.25	cr.	ins.	ins.	0.5	sol.
p-xylene	2.45	ins.	ins.		0.25	ins.	ins.	[A]	cr.
decalin	0.2	0.2	0.8	1.7	jelly	ins.	ins.	ins.	ins.
tetralin	3.0	1.8	jelly	0.5	0.2	sol.	ins.	jelly	4.0

^a^DMSO.

In many cases of chiral gelators, pure enantiomers were found more effective gelators than the racemates, although several exceptions were observed showing that the racemic form could be a more effective gelator of certain solvents than the corresponding pure enantiomer [60–68]. Therefore, we also compared gelation properties of selected enantiomers and racemates and found that the (*S,R*)-**1a**/(*R,S*)-**1a** racemate was considerably less effective in gelation of both, water/DMSO and water/DMF solvent mixtures compared to the pure enantiomer (*S,R*)-**1a**, while the racemate (*S,S*)-**1a**/(*R,R*)-**1a** lacked any gelation ability and tended to crystallize from both solvent mixtures.

Generally, it can be concluded that the retro-dipeptides are less effective and less versatile gelators compared to the previously studied bis(amino acid)oxalamides. Table 1 shows that bis(Leu)oxalamide **I** is much more versatile and a more efficient gelator compared to (*S,S*)-**1a** and (*S,R*)-**1a**, and is capable of gelling water and various solvents of medium and low polarity. However, **I** is considerably less efficient in gelation of highly polar water/DMSO and water/DMF solvent mixtures compared to both **1a** diasteroisomers. Hence, the presence of more hydrogen bonding sites and lipophilic groups in the retro-dipeptides appears less favorable for gelation of water and solvents of medium and low polarity presumably due to decreased solubility and increased crystallization tendency compared to bis(amino acid)oxalamides. However, their efficient gelation of water/DMSO and water/DMF solvent mixtures presents a striking difference where DMSO and DMF co-solvents could sufficiently increase their solubility up to the point necessary for aggregation into sufficiently long fibers capable of networking.

#### Terminal dimethyl ester retro-dipeptides

In the previously studied series of bis(amino acid)oxalamide gelators transformation of terminal carboxylic acid groups into methyl esters resulted in the complete loss of gelation ability [59]. In the retro-dipeptide series the gelation properties of methyl ester derivatives **1b**–**5b**, were not significantly different from those of the respective diacid derivatives **1a**–**5a** except that the diester derivatives appear slightly more versatile exhibiting gelation also with some lipophilic solvents (Table 1 and Table 2). This could be explained by the increased lipophilicity of the diester derivatives and, consequently increased solubility in more lipophilic solvents compared to the diacid gelators. It should be noted that the diester racemates (*S,R*)-**1b**/(*R,S*)-**1b** and (*S,S*)-**1b**/(*R,R*)-**1b** showed significantly increased effectiveness in gelation of water/DMSO and water/DMF solvent mixtures compared to the respective free acid racemates (*S,R*)-**1a**/(*R,S*)-**1a** and (*S,S*)-**1a**/(*R,R*)-**1a** (Table 1), respectively. Also, in contrast to the free acid gelators, the diester racemates were up to two times more efficient in the gelation of water/DMF and water/DMSO mixtures than their pure enantiomer counterparts (*S,R*)-**1b** and (*S,S*)-**1b** (Table 2). The latter provides additional examples that in some cases racemates could be more effective gelators than the pure enantiomers. Hence, in the search for highly effective gelators for targeted solvents, the racemic form of a chiral gelator must be tested.

**Table 2:** Gelator effectiveness (G_eff_, mL) of bis(dipeptide)oxalamide dimethyl esters **1b**–**5b** in gelation of various solvents and solvent mixtures (sol.: soluble; ins.: insoluble; cr.: crystallization; [A] gel/sol mixture; (F): cotton-like fiber aggregates; ** the mixture of crystals and gel.).

Solvent	(*S*,*S*)-**1b**	(*S,R*)-**1b**	(*S,R*)-**1b**/ (*R*,*S*)-**1b**	(*S*,*S*)-**1b**/ (*R,R*)-**1b**	**2b**	**3b**	**4b**	**5b**

H_2_O	ins.	ins.	ins.	ins.	ins.	ins.	ins.	ins.
H_2_O/DMSO	3.95+6.75	9.7+10.0	13.2+9.65	13.8+9.1	0.15+0.5	1.1+2.7	0.2+0.5	cr.
H_2_O/DMF	5.1+4.8	11.5+11.7	13.1+7.8	5.4+3.4	cr.	cr.	cr.	cr.
EtOH	cr.	cr.	cr.	cr.	cr.	2.00	(F1.3)	0.9
±2-octanol	cr.	cr.	cr.	cr.	1.85	cr.	(F0.5)	2.05
THF	sol.	cr.	sol.	sol.	sol.	sol.	sol.	cr.
CH_2_Cl_2_	sol.	sol.	sol.	sol.	sol.	ins.	sol.	cr.
CH_3_CN	cr.	cr.	cr.	cr.	cr.	cr.	cr	cr.
toluene	[A]	0.15	**	0.55	1.1	ins.	1.25	2.0
p-xylene	[A]	0.5	**	1.3	1.1	ins.	1.1	2.5
decalin	2.3	0.8	1.75	5.6	2.0	ins.	4.8	1.7
tetralin	cr.	0.2	sol.	sol.	0.6	0.4	0.9	0.5

#### Terminal dicarboxamide retro-dipeptides

The diamide derivatives bis(LeuLeuNH_2_) **1c**, bis(PhgPhgNH_2_) **3c** and bis(PhePheNH_2_) **5c** appeared more versatile being capable of gelling a larger set of tested solvents compared to the respective dicarboxylic acid (**1a**, **3a**, and **5a**) and dimethyl ester derivatives (**1b**, **3b** and **5b**) (Tables 1–3). The influence of stereochemistry on gelator versatility and effectiveness can be illustrated by the considerably improved gelation properties of the heterochiral (*S,R*)-**1c** diastereoisomer compared to homochiral (*S,S*)-**1c**; the former is capable of immobilizing up to 10 and 4 times larger volumes of dichloromethane and toluene solvent mixtures containing a little DMSO, respectively (Table 3). Also the bis(LeuPhgNH_2_) **2c** and bis(PhgLeuNH_2_) **4c** incorporating different amino acids appeared more versatile than the corresponding diacids (**2a**, **4a**) and diesters (**3b**, **4b**). It appears that the increased hydrogen bonding potential of terminal diamide derivatives provides somewhat more versatile gelators capable of gelating solvents of medium and low polarity where intermolecular hydrogen bonding is favored.

**Table 3:** Gelation effectiveness (G_eff_, mL) of bis(amino acid and dipeptide-CONH_2_)oxalamides **1c**–**5c** in gelation of various solvents and solvent mixtures (sol.: soluble, ins.: insoluble; cr.: crystalline; [A] gel/sol mixture).

Solvent	(*S*,*S*)**-1c**	(*S*,*R*)**-1c**	**2c**	**3c**	**4c**	**5c**

H_2_O	ins.	ins.	ins.	ins.	ins.	ins.
H_2_O/DMSO	0.55+1.4	0.8+1.2	1.8+2.9^a^	cr.	4.7+5.0	1.3+1.6
H_2_O/DMF	0.45+0.75	0.8+0.6	1.05+11^a^	cr.	1.7+1.8	2.7+3.0
EtOH	cr.	[A]	ins.	ins.	3.4	ins.
±2-octanol	ins.	3.0	5.0+0.1^a^	ins.	cr.	ins.
THF	ins.	ins.	1.1+0.05^a^	ins.	ins.	ins.
CH_2_Cl_2_	1.05+0.2^a^	11.0+0.84^a^	2.0+0.1^a^	ins.	1.5+0.4	ins.
CH_3_CN	ins.	0.5+0.04^a^	ins.	ins.	ins.	ins.
toluene	1.05+0.2^a^	4.0+0.4^a^	2.2+0.2^a^	ins.	0.75+0.1^a^	ins.
p-xylene	ins.	7.5+0.4^a^	6.9+0.2^a^	ins.	0.95+0.08^a^	ins.
decalin	ins.	ins.	0.8	ins.	ins.	0.5(ins.)
tetralin	7.3	sol.	2.0	ins.	1.0	2.0

^a^DMSO.

#### TEM and DSC investigations

**Figure 2:**
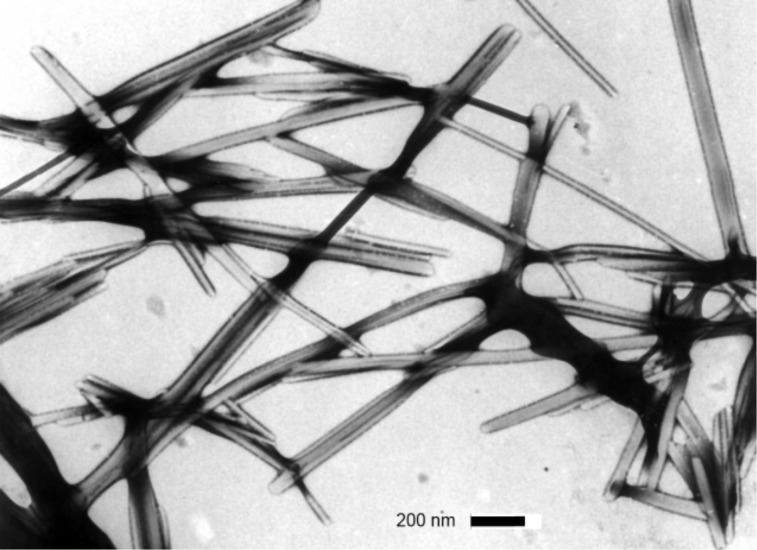
TEM images (PWK staining) of: (*S*,*S*)**-1a** H_2_O/DMSO gel.

**Figure 3:**
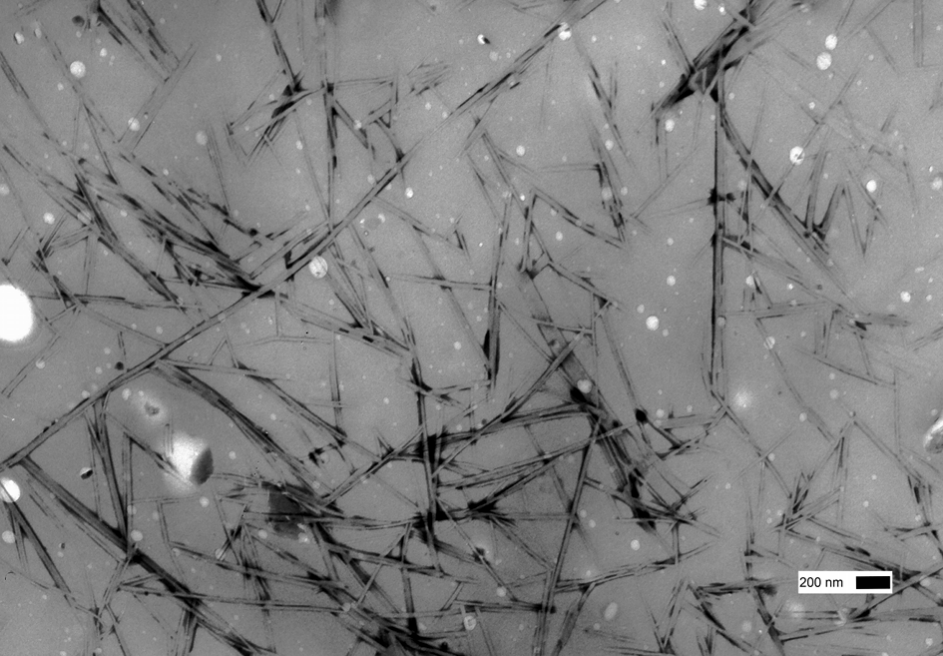
TEM images (PWK staining) of: (*S*,*R*)**-1a** H_2_O/DMSO gel.

**Figure 4:**
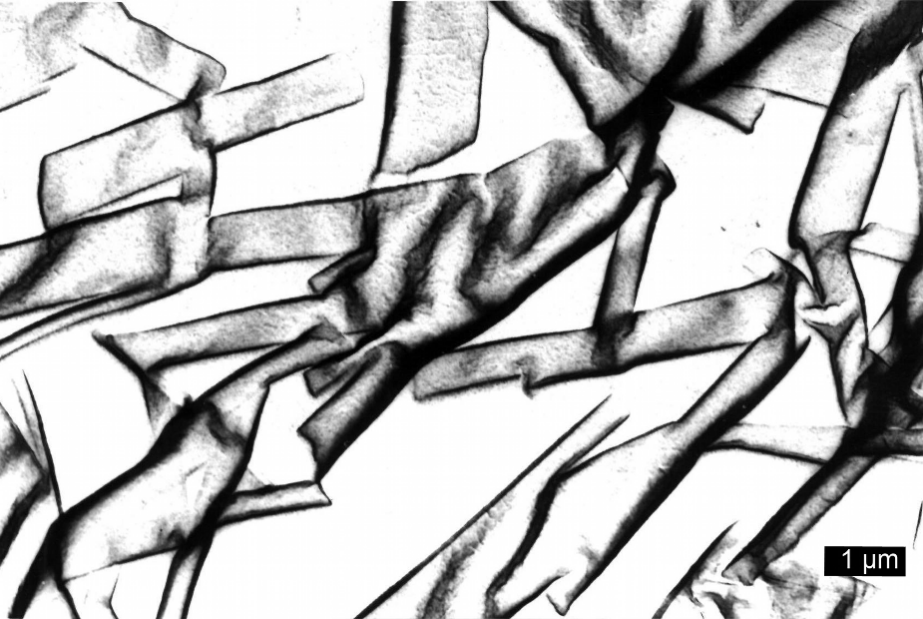
TEM images (PWK staining) of: (*S*,*R*)**-1b** toluene gel showing the presence of short tape like aggregates.

As reported previously, TEM investigations of bis(amino acid)oxalamide gels revealed in most cases formation of very dense networks consisting of heavily entangled tiny fibers with diameters in the range of 10–20 nm [58–60]. A similar morphology was observed for the bis(PhePhe)-**5a**-EtOH gel (fiber *d*’s 6–20 nm) and bis(PhgLeu) **4a** water/DMSO gel (fiber *d*’s 5–15 nm) (see Supporting Information File 1, Figure S1a,b). TEM images of diastereomeric (*S*,*S*)**-1a** and (*S*,*R*)**-1a** water/DMSO gels (Figure 2 and Figure 3) show highly distinct morphology of gel networks. In the first gel rather straight fibers and fiber bundles with diameters in the range of 40–100 nm could be observed. However, the (*S*,*R*)**-1a** network showed a lower bundling tendency (Figure 3) and contained mostly fibers with diameters between 20–40 nm. In contrast to water/DMSO gels, the TEM image of the methyl ester derivative (*S*,*R*)**-1b** gel with toluene had a totally different morphology characterized by the presence of short and very wide tapes (Figure 4). As observed earlier for other gel systems, gelator effectiveness G_eff_ depends not only solubility but also depends on the thickness of fibers constituting the network [43,59–60]. Since solvent is entrapped by capillary forces, the formation of a dense network composed of thin fibers should possess smaller compartments and hence a higher solvent immobilization capacity compared to those less dense formed by thick fibers. The TEM observed thicknesses of gel aggregates existing in water/DMSO and toluene gels could be also correlated with gelator effectiveness (G_eff_, mL, see Table 1 and Table 2). It appears that (*S*,*R*)**-1b** (G_eff_ 19.5 mL, water/DMSO gel) organized in thinner fibers is more than twice effective a gelator than its diasteroisomer (*S*,*S*)**-1a** (G_eff_ 8.0 mL, water/DMSO gel) which forms thicker fiber bundles. In the toluene gel, (*S*,*R*)**-1b** organizes into wide and short tapes with low networking capacity which is reflected in a very low (G_eff_ 0.15 mL) gelator effectiveness.

DSC investigation of the highly efficient (*S*,*R*)**-1a** gelator of water/DMSO solvent mixture showed only one transition in the heating (*T*_m_) and cooling (*T*_c_) cycle with gelation enthalpy changes of 37.70 and −38.20 kJ/mol, respectively (Table 4). The (*S*,*R*)**-1a/**(*R*,*S*)**-1a** racemic mixture being almost two times less effective in gelation of the same solvent mixture compared to (*S*,*R*)**-1a**, showed two transitions in the DCS heating and cooling cycle neither of which corresponded to those observed with (*S*,*R*)**-1a**. Moreover, the racemate showed considerably lower enthalpy changes compared to pure enantiomer gel (Table 4). The latter observations for the racemate gel indicate higher complexity of such systems and suggest possible interactions of enantiomers that lead to diasteromeric assemblies with a certain level of organization.

**Table 4:** Δ*H* and transition temperatures for selected retropeptide DMSO/water obtained from DSC heating and cooling cycles.

Gelator/solvent	*T*_m_	Δ*H*_m_	*T*_c_	Δ*H*_c_
	°C	kJ/mol	°C	kJ/mol

(*S*,*R*)-**1a/**water/DMSO	93.2	37.70	82.6	−38.20
(*S*,*R*)**-1a/**(*R*,*S*)**-1a/** water/DMSO	97.6 137.0	20.36 2.32	78.2 123.2	−23.95 −2.80

#### FTIR, ^1^H NMR and CD investigations

To identify supramolecular interactions that stabilize gel assemblies, the selected gels were studied by ^1^H NMR, FTIR and CD spectroscopy. Valuable information on the self-assembly of gelator molecules in the pre-gelation state and in the gel could be obtained by analysis of the concentration and temperature dependent ^1^H NMR and FTIR spectra. It was previously reported that the planar and self-complementary oxalamide unit persistently forms intermolecular hydrogen bonds and represents the major organizational element in the gel assemblies of both, bis(amino acid)- and bis(amino alcohol)oxalamides, and also has the major influence on their organization in the solid state [55–60]. In addition, the latter gelators tend to exhibit layered organization in their gel assemblies due to their structural resemblance to bola-amphiphiles.

In the FTIR spectra of (*S*,*S*)-**1b** and (*S*,*R*)-**1b** toluene gels one wide band or two poorly resolved NH bands, respectively, appear in the region of 3260–3320 cm^−1^ corresponding to a hydrogen bonded NH. In addition, the ester carbonyl and amide I bands are located at 1750 and 1653 cm^−1^, respectively, the position of the latter being in accord with its participation in hydrogen bonding.

**Figure 5:**
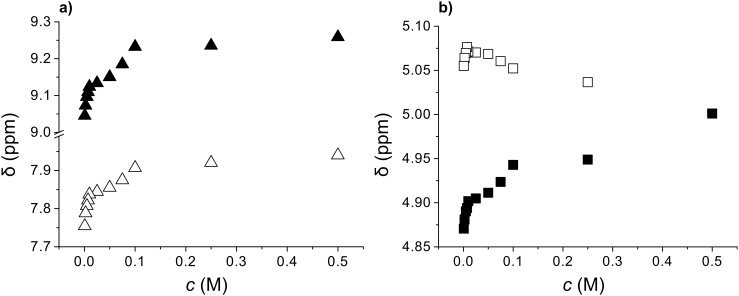
The concentration dependence of NH and C*H chemical shifts in (*S,R*)-**1b** toluene-*d*_8_ gel samples (concentration range 0.001–0.1 M): a) oxalamide-NH protons (▲); Leu-NH’s (Δ) and b) oxalamide-α-Leu-C*H (■) and oxalamide-β-Leu-C*H (□) protons.

**Figure 6:**
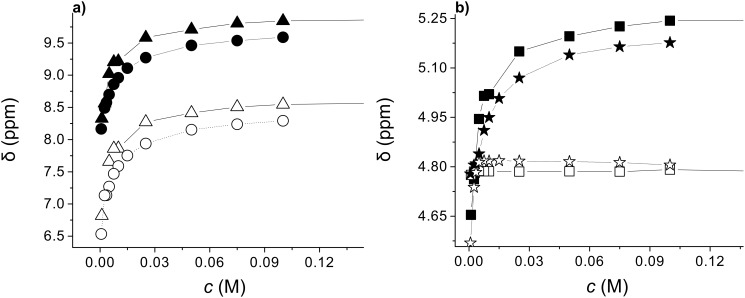
The concentration dependence of NH and C*H chemical shifts in (*S,S*)-**1b** and its racemate (*S,S*)-**1b**/(*R,R*)-**1b** toluene-*d*_8_ gels (concentration range 0.001–0.1 mol dm^−3^): a) (*S,S*)-**1b** oxalamide-NH protons (▲) and Leu-NH protons (Δ); the racemate oxalamide-NH protons (●) and Leu-NH protons (O); b) (*S,S*)-**1b** oxalamide-α-Leu-C*H (■);(*S,S*)-**1b** oxalamide-β-Leu-C*H (□); the racemate oxalamide-α-Leu-C*H (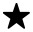); racemate oxalamide-β-Leu-C*H (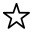).

^1^H NMR investigation showed significant concentration dependence of N–H and C*H proton shifts in the (*S*,*R*)-**1b** (Figure 5), (*S,S*)-**1b** and its racemate (*S,S*)-**1b**/(*R,R*)-**1b** (Figure 6) toluene-*d*_8_ gel samples. In the first case the oxalamide NH and Leu-NH protons were downfield shifted by 1.6 and 1.8 ppm, respectively, for a gelator concentration increase from 0.001–0.1 mol dm^−3^. The oxalamide-α-Leu methine protons (C*H) were also significantly downfield shifted (Δδ_C*H_ = 0.566 ppm) while the β-Leu-C*H proton shifts were less significant. Strong downfield shifts of the oxalamide- and Leu-NH protons as well as the α-Leu-C*H proton closest to the oxalamide unit suggest simultaneous participation of both the oxalamide and Leu-NH protons in intermolecular hydrogen bonding. A comparison of the magnitudes of concentration induced shifts for diastereomeric gelators (*S*,*R*)-**1b** (Figure 5; oxalamide-NH protons Δδ 0.25 ppm; Leu-NH protons Δδ 0.17 ppm; α-Leu-C*H Δδ 0.13 ppm) and (*S,S*)-**1b** (Figure 6, oxalamide-NH protons Δδ 1.50 ppm; Leu-NH protons Δδ 1.55 ppm; α-Leu-C*H Δδ 0.53 ppm) shows large differences. The monitored protons of (*S,S*)-**1b** are more strongly downfield shifted than the corresponding protons of (*S*,*R*)-**1b** for the same concentration range. Similar trends of NH and C*H concentration induced shifts are observed for (*S,S*)-**1b** and its racemate (*S,S*)-**1b**/(*R,R*)-**1b** toluene-*d*_8_ gels (Figure 6). Again the magnitudes of the NH and C*H concentration induced shifts are higher for the (*S,S*)-**1b** than for the racemate gel. It should be noted that the higher concentration induced shifts are observed for (*S,S*)-**1b** which forms sol–gel mixture in toluene compared to both (*S*,*R*)-**1b** and the racemate (*S,S*)-**1b**/(*R,R*)-**1b** forming stable toluene gels (Table 2).

The concentration induced shift curves show that for the examined gelators, the self-assembly equilibrium is reached at different gelator concentrations; for (*S,S*)-**1b** and its racemate (*S,S*)-**1b**/(*R,R*)-**1b**, the saturation point is reached at the same concentration of 0.03 mol dm^−3^ (Figure 6a,b) which corresponds to the experimentally determined minimal gelation concentration (*MGC*) for the racemate of 0.034 mol dm^−3^. If the racemate were organized in the conglomerate as separate enantiomeric (*S,S*)-**1b** and (*R,R*)-**1b** assemblies, the magnitudes of the concentration induced shifts should be similar to those measured the for (*S,S*)-**1b** assemblies. Since this was not observed (Figure 6a, b) the results suggest formation of racemic gel assemblies composed of both enantiomers. The latter also points to the lack of any resolution at the supramolecular level which was found to occur for some racemic gelators and specific solvents [60]. The observation that (*S,S*)-**1b** with toluene gives a sol/gel mixture while the racemate gives a stable gel implies that the enantiomer forms insufficiently long assemblies incapable of efficient networking and of forming of self-supported gel, while the opposite holds for the racemic assemblies which are capable of forming the gel network

The discontinuous concentration induced shift curves obtained for (*S,R*)-**1b** diasteroisomer may indicate the presence of different assemblies at lower and higher gelator concentrations. It appears that the first saturation point is reached at a concentration around 0.03 mol dm^-3^ and the second at 0.12 mol dm^−3^, the latter corresponding nicely to the experimentally determined *MGC* of 0.116 mol dm^−3^. It should be noted that in the low and high concentration ranges downfield shifts of oxalamide- and Leu-NH protons are observed indicating that both assemblies are formed by intermolecular hydrogen bonding. The determined higher saturation point and *MGC* of 0.12 mol dm^−3^ for (*S,R*)-**1b** compared to the saturation point of the (*S,S*)-**1b** diastereoisomer (0.03 mol dm^−3^) could be explained by the increased solubility of the (*S,R*)-**1b** assemblies in toluene compared to those formed by the second diastereoisomer [69]. However, despite of the lower saturation point and lower solubility, the (*S,S*)-**1b** assemblies cannot form the gel which points toward possible solvation effects taking a decisive role in the self-assembly of the diastereoisomers. Recently, Meijer et al. [70] presented convincing evidence that co-organization of solvent at the periphery of the gel aggregates plays a direct role in the assembly processes evident, even during the formation of the pre-aggregates. The influence of solvent structure on the length of the aggregates was clearly demonstrated. Hence, different solvation effects of toluene operating in the self-assembly of (*S,S*)-**1b** and (*S,R*)-**1b** may be responsible for the formation of insufficiently long aggregates of the first diastereoisomer resulting in the formation of the sol–gel mixture, and sufficiently long assemblies of the second one being capable of networking and the formation of a self-supported gel.

**Figure 7:**
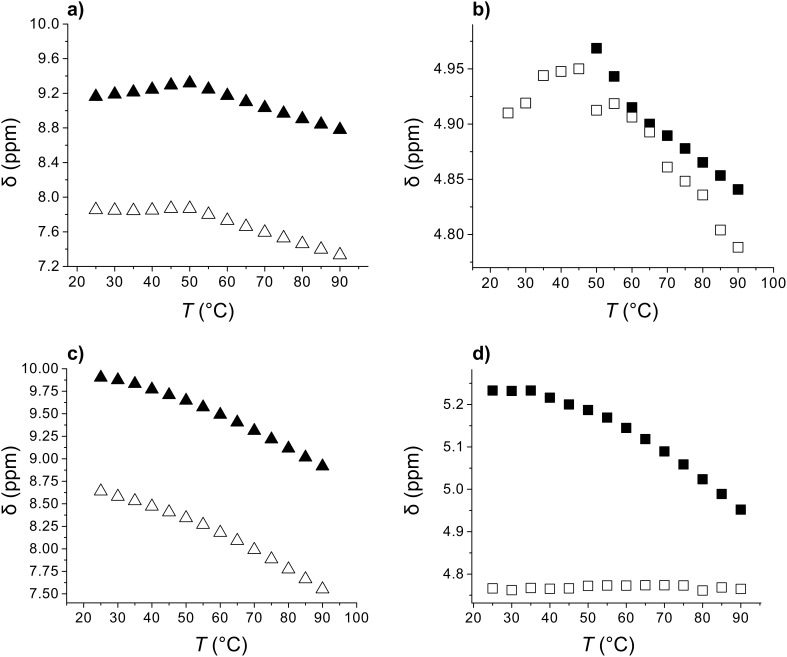
Temperature dependence of: a) oxalamide NH protons (▲), Leu-NH protons (Δ) and b) oxalamide-α-Leu-C*H (■) and oxalamide-β-Leu-C*H (□) chemical shifts in 0.5 mol dm^−3^ (*S,R*)-**1b** toluene-*d*_8_ gel sample; c) and d) induced shifts of the respective protons in (*S,S*)-**1b** toluene-*d*_8_ gel.

The variations of oxalamide NH, Leu-NH, α- and β-Leu-C*H proton chemical shifts with increasing temperature in the toluene-*d*_8_ gel samples of the diastereomeric (*S,R*)-**1b** and (*S,S*)-**1b** (concentrations of 0.5 mol dm^−3^) are shown in Figure 7. For the (*S,R*)-**1b** gel, a temperature increase from 20–50 °C induced only slight downfield shifts of both the oxalamide- and Leu-NH protons as well as the α- and β-Leu-C*H protons; in the higher temperature interval (50–90 °C) all protons were downfield shifted in accord with the breaking of intermolecular hydrogen bonds involving both the oxalamide and Leu NH protons. In contrast, the respective protons of the (*S*,*S*)-**1b** are continuously shifted downfield with increasing temperature (Figure 7c). Hence, a clear difference in the thermal behavior of (*S,S*)-**1b** weak gel and (*S,R*)-**1b** gels was observed. Similar discontinuous temperature variation curves to those observed for (*S,R*)-**1b** were also found for bis(amino acid)oxalamide gelators which were shown to exhibit the layered type of organization in their gel assemblies [59–60]. Small downfield shifts of the oxalamide- and LeuNH protons observable in the low temperature regime (Figure 7a) were explained by the less energy demanding disassembly that occurred at lipophilic sites of the interacting bilayers resulting in small deshielding of these protons. In the higher temperature regime the downfield shifts of the same protons indicate the breaking of intermolecular hydrogen bonds. This conclusion is supported by molecular modeling (see the respective paragraph) which showed that the low energy conformation of (*S,R*)-**1b** is similar to those found for bis(amino acid)oxalamides and that both show a strong resemblance to bola-amphiphiles which are known to organize into bilayers [71].

**Figure 8:**
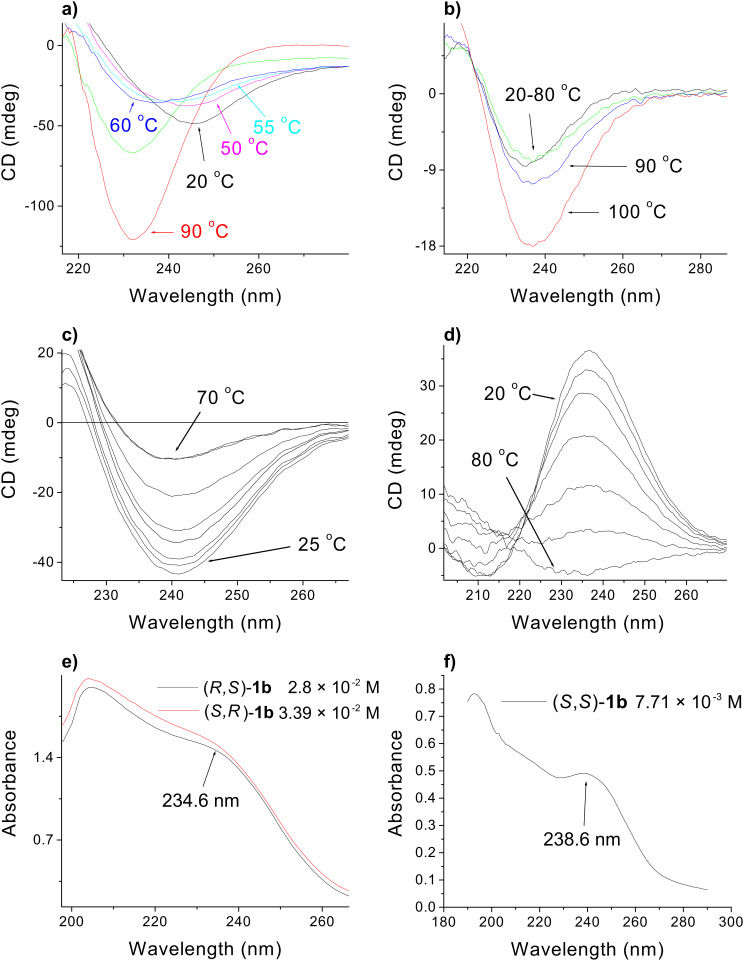
Temperature dependent CD spectra of: a) (*S,R*)-**1b** decalin gel (*c* = 3.4·10^−2^ M); b) (*S,S*)-**1b** decalin gel (*c* =7.6·10^−3^ M); c) **5a** ethanol gel (*c* =1·10-2 M) and d) (*S,S*)-bis(Leu)oxalamide **I** 1-butanol gel (*c* = 2.8·10^−2^ M); e), f) UV spectra of (*S,R*)-**1b** (red curve), (*R,S*)-**1b** and (*S,S*)-**1b** taken in decalin at specified concentrations.

The temperature dependence of CD spectra of decalin gels formed by diastereoisomeric methyl esters (*S,R*)-**1b** and (*S*,*S*)-**1b** (Figure 8a, b, respectively) was also investigated. At room temperature (*S,R*)-**1b** shows negative Cotton effect at λ = 245 nm of moderate intensity which decreases on increasing temperature from 20 to 50 °C. Further temperature increase of the gel sample resulted in the appearance of a new negative CD peak at λ = 234 nm corresponding to the shoulder band in the gelator UV spectrum (Figure 8e); the intensity of the band increased with increasing temperature (Figure 8a). As reported for the self-assembled alanine based gelators, the CD signal at around 232 nm can be ascribed to the n,π^*^-transition of the amide carbonyl [72–74]. Hence, the λ = 234 nm band that appeared at 60 °C could be ascribed to the intrinsic chirality of disassembled gelator molecules. Although the origin of the λ = 245 nm CD band is not clear, it could be the consequence of circular differential scattering which was shown to contribute to the CD spectra of large aggregated biomolecules [75].

By contrast, the CD spectra of the (*S*,*S*)-**1b** gel showed a negative CD band at λ*_min_* 238.6 nm (Figure 8b) corresponding to its electronic absorption band (Figure 8f), but similarly to (*S,R*)-**1b** the intensity of CD band increased with increasing temperature.

It should be noted that the temperature induced changes in the CD spectra of both, (*S,R*)-**1b** and (*S*,*S*)-**1b** decalin gels are different to those obtained for **5a** ethanol and bis(Leu)oxalamide 1-butanol gels (Figure 8c, d). With these latter gels a decrease of CD peak intensities with increasing temperature was observed in accord with the disassembly of the chiral gel aggregates which has also been observed for some other chiral gels [76]. Consequently, the CD results described for the (*S,R*)-**1b** and (*S*,*S*)-**1b** gels, characterized by the increase of CD signals with increasing gel temperature, indicate that in these systems there is no aggregation increased chirality as observed for some other gels of the chiral gelators.

#### XRPD, molecular modeling and packing model

**Figure 9:**
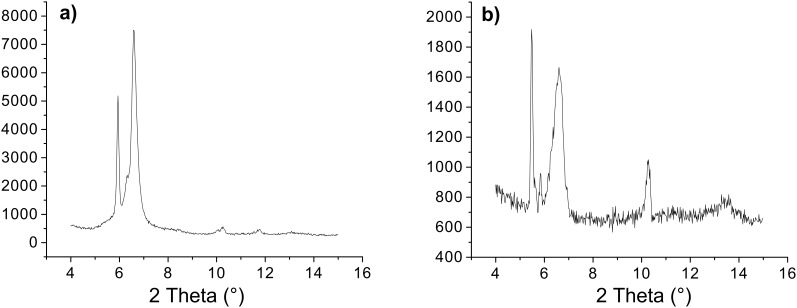
X-ray powder diffractograms of (a) (*S,R*)-**1b** and (b) (*S,S*)-**1b** xerogels prepared from their toluene gels.

The X-ray powder diffraction (XRPD) pattern of (*S,S*)-**1b** xerogel showed strong peaks corresponding to periodic distance *d* of 16.1 and 13.4 Å and a weaker peaks corresponding to *d’s* of 15.1 and 8.6 Å (Figure 9b).

**Figure 10:**
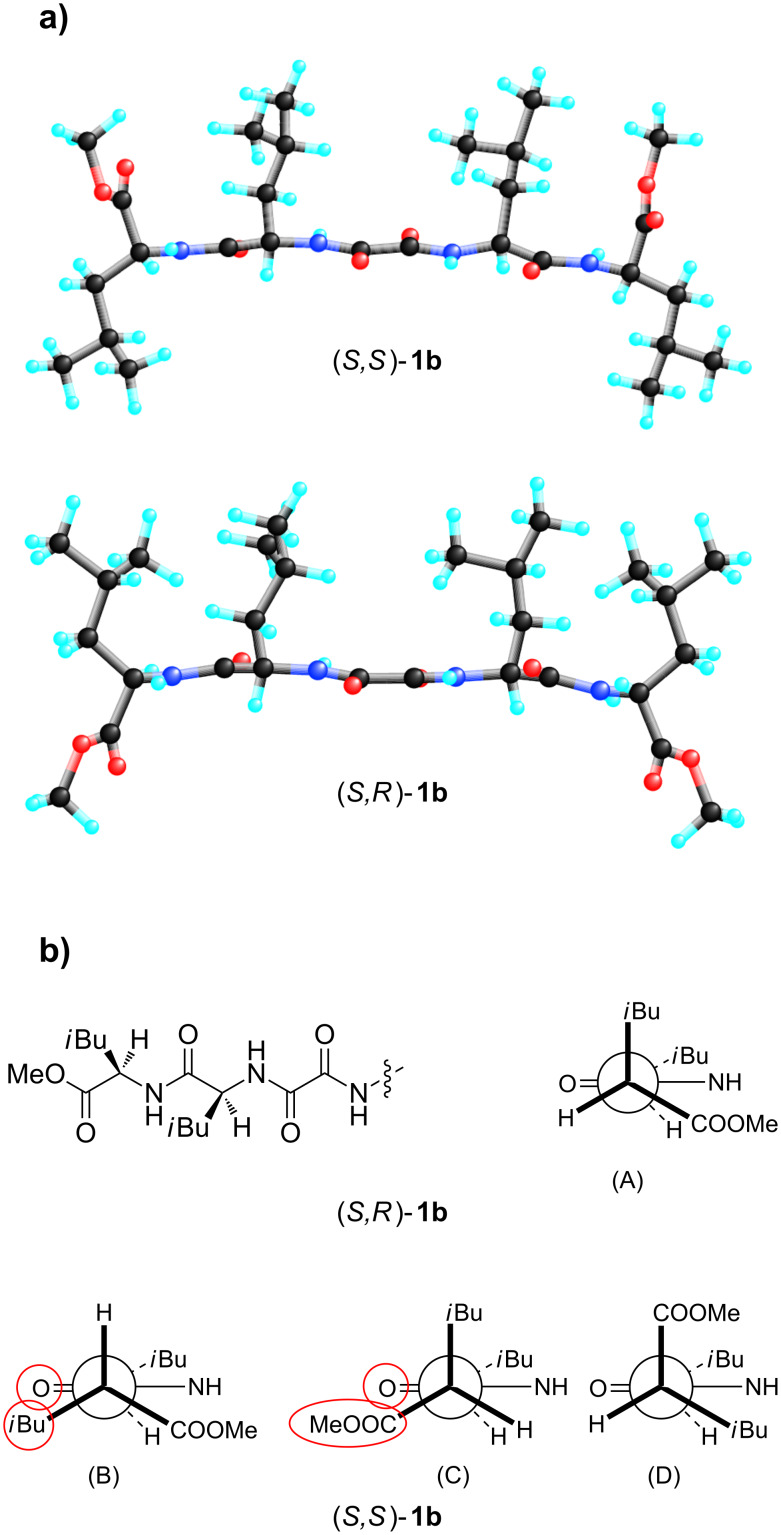
(a) Fully minimized the lowest energy conformations of (*S,S*)-**1b** (top) and (*S,R*)-**1b** generated by systematic conformational search (SYBYL package; second graphic); (b) Partial Newman projections of two stereogenic centers of (*S,R*)-**1b** and (*S,S*)-**1b** showing conformations with *cis*-arrangement of *i*-Bu groups in the former (A) and *trans*- (B, D) and *cis*- (C)-arrangements of *i*-Bu groups in the latter.

For the (*S*,*R*)-**1b** xerogel, strong peaks corresponding to *d*’s of 14.9 and 13.4 Å and smaller peaks to *d*’s of 13.98, 8.6 and 7.5 Å could be observed (Figure 9a). Molecular modeling of (*S,R*)-**1b** and (*S*,*S*)-**1b** yields low energy extended conformations with lengths of 15.1 and 15.9 Å, respectively (Figure 10a) [77].

The measured extended conformation lengths correspond nicely to the largest periodic distances of 14.9 and 16.1 Å obtained by XRP diffraction of (*S,R*)-**1b** and (*S*,*S*)-**1b** xerogels indicative of the formation of assemblies of the extended gelator molecules. However, the low energy conformations of (*S,R*)-**1b** and (*S*,*S*)-**1b** are distinctly different with all *i-*Bu groups *cis-*oriented in (*S,R*)-**1b**, while the *i-*Bu groups in α- and β-Leu of (*S*,*S*)-**1b** have the *trans*-arrangement with respect to the plane containing the amide and oxalamide groups (Figure 10a). Conformational analysis reveals why such arrangements of *i-*Bu groups occur (Figure 10b, Newman projections of two stereogenic centers only). Our earlier results based on single crystal X-ray analysis of bis(amino acid)oxalamides showed that their most stable conformations are characterized by vicinal positioning of the methine proton at the stereogenic centre and oxalamide carbonyl oxygen atom which produces the lowest steric repulsion. Similarly, in the conformation A of (*S,R*)-**1b** with *cis*-arrangement of the *i-*Bu groups, the smallest group (H) of the β-Leu chiral centre is located close to amide carbonyl; the conformations with *trans*-arrangement of *i-*Bu groups should be less stable due to increased steric repulsion between the amide carbonyl oxygen and either the *i-*Bu or carboxymethyl group. Among the conformations of (*S,S*)-**1b** denoted B, C and D with *trans*-, *cis*- and *trans*-arrangement of *i-*Bu groups, respectively, the conformation D appears the most stable due to the vicinal position of the smallest group (H) and amide carbonyl oxygen atom. These conclusions are supported by molecular modeling (Figure 10a); the lowest energy conformations of (*S,R*)-**1b** and (*S*,*S*)-**1b** generated by systematic search of their conformational space correspond to A and D of Figure 10b, respectively. In support, the values of the vicinal NH-Cα-H coupling constants *J_NH-CH_* for the (*S,R*)-**1b** oxalamide NH-Leu Cα-H and Leu NH-Leu Cα-H (8.63 and 8.48 Hz) and (*S*,*S*)-**1b** (8.63 and 8.33 Hz) obtained from their ^1^H NMR spectra taken in CDCl_3_ correspond to dihedral angles close to *trans*-coplanar positioning of NH and Cα-H protons in both groups

**Figure 11:**
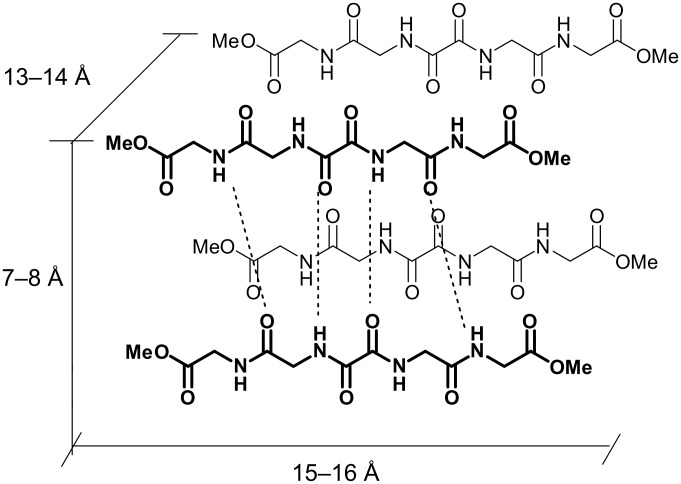
Schematic presentation of the proposed (*S,S*)-**1b** and (*S,R*)-**1b** basic packing model based on XRPD, ^1^H NMR, FTIR and molecular modeling results.

The low energy conformations of (*S,R*)-**1b** and (*S*,*S*)-**1b** were used for docking calculations to generate the hydrogen bonded dimers of extended gelator molecules involving both, the oxalamide and Leu-NH protons (Supporting Information File 1, Figure S3). The thicknesses of such dimers estimated from models are between 7.5 and 8.6 Å (Figure 11) which correspond well to periodic distance *d* of 8.6 Å found in their XRP diffractograms. The thickness of the (*S,R*)-**1b** dimer generated by lipophilic interactions is 13.4 Å corresponding exactly to *d* of 13.4 Å found in its XRPD. The (*S*,*S*)-**1b** model of the dimer formed by lipophilic packing gives a thickness of 13.9 Å. Based on these results and those of the FTIR and ^1^H NMR studies, which suggested intermolecular hydrogen bonding between gelator molecules involving both the oxalamide and Leu amide units, a basic packing model for (*S*,*S*)-**1b** and (*S,R*)-**1b** can be proposed which consists of layers of hydrogen bonded gelator molecules (Figure 11).

In contrast to the diester gelators (*S,R*)-**1b** and (*S*,*S*)-**1b** a detailed spectroscopic investigation of (*S,S*)-**1a** and (*S,R*)-**1a** organization in their water/DMSO gel assemblies was not possible due to solvent unsuitability.

**Figure 12:**
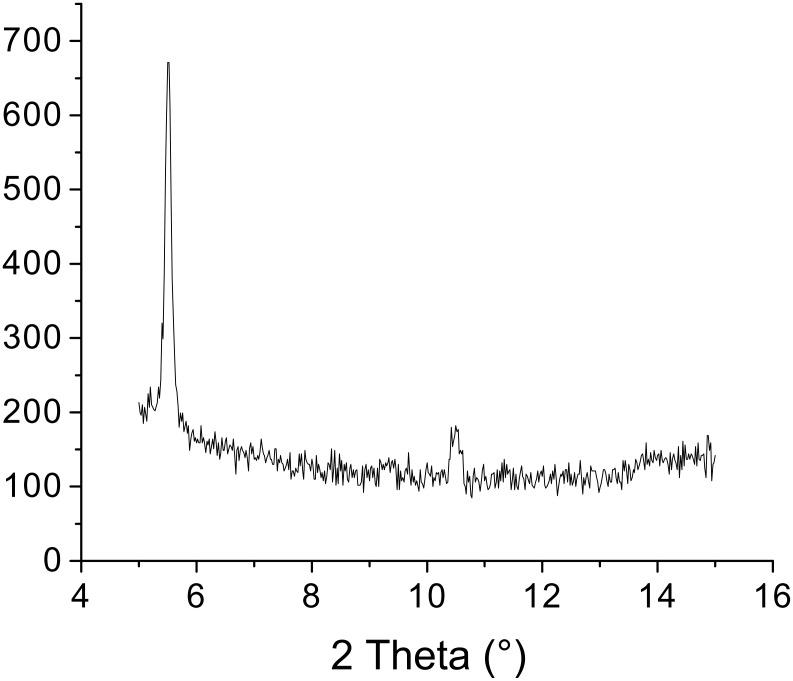
X-ray powder diffraction (XRPD) diagram of (*S,R*)-**1a** water/DMSO xerogel.

Nevertheless, the FTIR spectrum of the (*S,R*)-**1b** xerogel prepared from its water/DMSO gel was found to differ from that of the crystalline sample; the positions of NH stretching, carboxylic acid and amide I carbonyl stretching, and NH bending amide II bands in the spectra of crystalline and xerogel samples appear at 3281.2 1724.4 1655.6 1543.6 1510.4 cm^−1^ and 3303.4 3273.5 1728.5 1651.6 1534.2 1510.5 cm^−1^, respectively. The positions of the xerogel bands are similar to those found in the spectrum of previously studied bis(Leu)oxalamide water/DMSO gel assemblies (3300 1729 1658 1515 cm^−1^) and which was shown to organize by intermolecular hydrogen bonding between oxalamide units and lateral carboxylic acid hydrogen bonding [59]. The appearance of two NH stretching and amide II bands in the (*S,R*)-**1b** xerogel spectrum (3303.4 3273.5 1534.2 1510 cm^−1^) can be attributed to the intermolecular hydrogen bonds formed by Leu amide units. The XRPD of (*S,R*)-**1a** water/DMSO gel (Figure 12) showed two diffraction peaks at 2θ = 5.509 and 10.501 corresponding to periodic distances *d* of 16.04 and 8.42 Å which also suggests formation of hydrogen bonded assemblies between extended forms of gelator molecules as in the cases of the diester derivatives (*S,S*)-**1b** and (*S,R*)-**1b** (Figure 11).

These results indicate that the dicarboxylic retro-dipeptides (*S,S*)-**1a** and (*S,R*)-**1a** also show similar basic organization as their dimethyl ester counterparts (*S,S*)-**1b** and (*S,R*)-**1b** (Figure 11). Also molecular modeling of (*S,S*)-**1a** and (*S,R*)-**1a** generated very similar low energy conformations to those of (*S,S*)-**1b** and (*S,R*)-**1b** shown in Figure 10a, b. In both cases, the major organizational driving force is provided by extensive intermolecular hydrogen bonding. In lipophilic solvents, where such types of intermolecular interactions are highly favored, formation of wide and relatively short tapes could be observed (TEM, (*S,R*)-**1b** toluene gel, Figure 4) possibly due to the enhanced self-assembling in the direction of intermolecular hydrogen bonds. In contrast, gelling of the highly polar and hydrogen bond competitive water/DMSO solvent mixture (TEM, (*S,S*)-**1a** and (*S,R*)-**1a** water/DMSO gels, Figure 2 and Figure 3) results in the formation of tiny fibers or fiber bundles due to less favored self-assembly in the direction of intermolecular hydrogen bonding and more pronounced intermolecular lipophilic interactions.

## Conclusion

A series of chiral bis(dipeptide)oxalamides was prepared representing a novel family of retro-peptidic gelators. Their gelation properties towards a defined set of solvents was assessed and, their conformational characteristics, organization in gel assemblies and thermal and morphological characteristics of selected gels were studied by molecular modeling, ^1^H NMR, FTIR, CD, DCS, TEM and XRPD. Gelation experiments have shown that the group of terminal free acid retro-dipeptides (*S*,*S*)-bis(LeuLeu) **1a**, (*S,S*)-bis(PhgPhg) **3a** and (*S,S*)-bis(PhePhe) **5a** showed moderate to excellent gelation of polar water/DMSO and water/DMF solvent mixtures and were much less efficient in gelating solvents of medium and low polarity. Interestingly, the free acid gelators incorporating different amino acids (*S,S*)-(LeuPhg) **2a** and (*S,S*)-(PhgLeu) **4a** had no or very weak gelating ability. The observed difference in gelation between retro-peptides incorporating identical or two different amino acids is intriguing. It seems that the intermolecular lipophilic interactions that stabilize gel assemblies in polar solvents are more favorable for identical amino acid fragments (lipophilic or aromatic) and less favorable when aromatic–lipophilic amino acid fragments are present in the gelator molecule. Gelation properties of methyl ester derivatives **1b**–**5b**, were not significantly different to those of the respective diacid derivatives **1a**–**5a** except that the former appear slightly more versatile and were capable of gelating some lipophilic solvents presumably due to increased solubility (Table 1 and Table 2). The diamide derivatives bis(LeuLeuNH_2_) **1c**, bis(PhgPhgNH_2_) **3c** and bis(PhePheNH_2_) **5c** were even more versatile and were capable of gelating a larger set of tested solvents compared to the respective dicarboxylic acid (**1a**, **3a**, and **5a**) and dimethyl ester derivatives (**1b**, **3b** and **5b**) (Tables 1–3). It appears that the increased hydrogen bonding potential of terminal diamide derivatives gives somewhat more versatile gelators which could also gel solvents of medium and low polarity where intermolecular hydrogen bonding is favored. Stereochemical influences on gelation properties are exemplified by the following observations: (i) the racemate (*S,R*)-**1a**/(*R,S*)-**1a** exhibited considerably lower gelation effectiveness than the pure enantiomer (*S,R*)-**1a** while the (*S,S*)-**1a**/(*R,R*)-**1a** racemate had no gelation ability and tended to crystallize, (ii) terminal diester racemates (*S,R*)-**1b**/(*R,S*)-**1b** and (*S,S*)-**1b**/(*R,R*)-**1b** were two times more efficient in the gelation of water/DMF and water/DMSO mixtures, respectively, than the pure enantiomers (*S,R*)-**1b** and (*S,S*)-**1b**; the latter provides additional examples that some racemates could be more effective gelators of specific solvents than the pure enantiomers; (iii) among the terminal carboxamide gelators the heterochiral (*S,R*)-**1c** diastereoisomer is capable of immobilizing up to 10 and 4 times larger volumes of dichloromethane and toluene solvent mixtures containing a little DMSO, respectively, compared to homochiral (*S,S*)-**1c**. The combined results of ^1^H NMR, FTIR, XRPD and molecular modeling studies of terminal diester (*S,S*)-**1b** and (*S*,*R*)-**1b** and terminal free acid (*S,S*)-**1a** and (*S*,*R*)-**1a** derivatives gave a consistent picture of their basic organization in gel assemblies. In lipophilic solvents and also in the highly polar water/DMSO mixture, the intermolecular hydrogen bonding between extended gelator molecules utilizing both, the oxalamide and Leu amide hydrogen bonding functionalities represents the major organizational driving force for aggregation. The TEM investigations have shown that in the highly lipophilic solvents the extensive intermolecular hydrogen bonding may lead to the formation of wide and relatively short tapes giving a gel network of low solvent immobilization capacity. Molecular modeling studies revealed that the homochiral (*S,S*)-**1a** and (*S,S*)-**1b** form the low energy conformations with *cis*-*trans*-arrangement of *i*-Bu groups in contrast to the heterochiral (*S*,*R*)-**1a** and (*S*,*R*)-**1b** conformations possessing the all-*cis*-arrangement of *i*-Bu groups with respect to the oxalamide plane. Such conformational differences were found to strongly influence both, gelation effectiveness and the morphology characteristics of gel network.

## Supporting Information

File 1 Full experimental procedures and characterization details for all new compounds, molecular modeling and TEM images.
