# Supplementary material for: Oxalyl retro-peptide gelators. Synthesis, gelation properties and stereochemical effects
Source: Beilstein J Org Chem. 2010 Oct 4;6:945–59. doi: 10.3762/bjoc.6.106 (PMC2981816; doi:10.3762/bjoc.6.106)
Supplement: File 1 — Full experimental procedures and characterization details for all new compounds, molecular modeling and TEM images. [file Beilstein_J_Org_Chem-06-945-s001.pdf]

Supporting Information

for

Oxalyl retro-peptide gelators. Synthesis, gelation properties and stereochemical effects

Janja Makarević, Milan Jokić, Leo Frkanec, Vesna Čaplar, Nataša Šijaković Vujičić, and Mladen Žinić*

Laboratory for Supramolecular and Nucleoside Chemistry, Ruđer Bošković Institute, P.O.B. 180, HR-10002 Zagreb, Croatia

Email: Mladen Žinić - zinic@irb.hr

* Corresponding author

Full experimental procedures and characterization details for all new compounds, molecular modelling and TEM images

Experimental Section

General

Melting points were determined on Kofler hot stage and are uncorrected. ^{1}H and ^{13}C NMR spectra were recorded at 300 and 75 MHz, respectively, on a Gemini 300 spectrometer (TMS was used as internal standard). FTIR experiments on gels were performed in sealed heatable cells for liquids (path length 0.05 mm, CaF_2 windows) and recorded on an ABB Bomen MB 102 FTIR-spectrometer. UV–vis

measurements were carried out on a Cary 5 and PU8730 UV/VIS spectrophotometer. Optical rotations were measured on an AA-10 automatic polarimeter at a wavelength of 589.3 nm. TLC was performed on silica gel coated Merck 60 silica plates and column chromatography carried out with 230–240 mesh Merck 60 silica gel. All chemicals were of the best grade commercially available and were used without purification. Solvents were purified according to standard procedures; dry solvents were obtained according to literature methods and stored over molecular sieves.

***General procedure for the preparation of oxalamide - diesters* 1b–5b**

To a cooled (-10 °C) solution of H-A_{aa}-B_{aa}-OMe **1 – 5** (1.1 mmol) in dry CH_2Cl_2 (30 mL) and TEA (2.1 mmol), a solution of oxalyl chloride (0.5 mmol) in CH_2Cl_2 (10 mL) was added. The mixture was stirred at 0 °C for 30 min and then overnight at room temperature. The reaction mixture was then washed successively with 5 % HCl, 5 % $NaHCO_3$ and water. The organic layer was dried (Na_2SO_4) and the solvent evaporated under reduced pressure.

(*S,S*)-*N,N'*-Oxalyl-bis(leucyl-leucine methyl ester) (1b)[4]; *Following the general procedure the title compound was obtained starting from* (*S,S*)-Leu-Leu-OMe. The product was purified by preparative TLC (CH_2Cl_2:MeOH 30:1); yield 78 %; M.p. 208–211 °C (from CH_2Cl_2–light petroleum) (1it[4] 200–201 °C); $[\alpha]_D^{20} = -68$ (*c* 1 in CH_2Cl_2); ^{1}H NMR (300 MHz, $CDCl_3$, 20 °C, TMS): δ = 8.11 (d, $^3J_{H,H}$ = 9.0 Hz, 2 H, NH), 6.87 (d, $^3J_{H,H}$ = 8.1 Hz, 2 H, NH), 4.58–4.51 (m, 4 H, CH_α), 3.71 (s, 6 H, $CH_{3\ (OMe)}$), 1.80–1.50 (m, 12 H, $CH_{2\ (\beta)}$ and CH_γ), 0.92–0.85 (m, 24 H, $CH_{3\ (\delta)}$); ^{13}C NMR (75.5 MHz, $CDCl_3$, 20 °C): δ = 173.3 and 170.7 (CONH and COOMe), 159.2 ($CONH_{oxal}$), 52.0 ($CH_{3\ (OMe)}$), 51.8 and 50.6 (CH_α), 40.9 and 40.5 ($CH_{2\ (\beta)}$), 24.5 and 24.4 (CH_γ), 22.6, 22.34, 22.29, 22.0, 21.8 and 21.7 ($CH_{3\ (\delta)}$); IR

(KBr): ν = 3400 br (NH), 3315 br and 3270 (NH), 1750 (COOMe), 1659 and 1652 (amide I), 1540 and 1518 (amide II) cm^{-1}.

(*S,R*)-*N,N′*-Oxalyl-bis(leucyl-leucine methyl ester) 1b and **(*R,S*)-*N,N′*-Oxalyl-bis(leucyl-leucine methyl ester) 1b** were prepared starting from (*S,R*)- and (*R,S*)-Leu-Leu-OMe, repectively following the same procedure described above.

(*S,S*)-*N,N′*-Oxalyl-*bis*(leucyl-phenylglycine methyl ester) (2b); *Following the general procedure the title compound was obtained starting from* **(*S,S*)**-Leu-PhgOMe, yield: 75.8 %; M.p. 217–222 °C (from CH_2Cl_2–light petroleum); $[\alpha]_D^{20}$ = +84 (*c* 0.5 in CH_2Cl_2); ^{1}H NMR (300 MHz, $CDCl_3$, 20 °C, TMS): δ = 8.13 (d, $^3J_{H,H}$ = 8.9 Hz, 2 H, NH_{Leu}), 7.71 (d, $^3J_{H,H}$ = 7.3 Hz, 2 H, NH_{Phg}), 7.18–7.10 (m, 10 H, H_{arom}), 5.48 (d, $^3J_{H,H}$ = 7.3 Hz, 2 H, $CH_{\alpha, Phg}$), 4.64 (dt, $^3J_{H,H}$ = 6.2 Hz and $^3J_{H,H}$ = 8.9 Hz, 2 H, $CH_{\alpha, Leu}$), 3.61 (s, 6 H, $CH_{3\,(OMe)}$), 1.80–1.60 (m, 6 H, $CH_{2\,(\beta, Leu)}$ and $CH_{\gamma\,(Leu)}$), 0.92 and 0.88 (2 d, $^3J_{H,H}$ = 6.2 Hz, 6 H each, $CH_{3\,(\delta, Leu)}$); ^{13}C NMR (75.5 MHz, $CDCl_3$, 20 °C, TMS): δ = 171.2 and 170.2 (CONH and COOMe), 159.1 ($CONH_{oxal}$), 135.7, 128.8, 128.3 and 127.0 (C_{arom}), 56.0 ($CH_{\alpha, Phg}$), 52.5 ($CH_{3\,(OMe)}$), 51.8 ($CH_{(\alpha, Leu)}$), 40.7 ($CH_{2\,(\beta, Leu)}$), 24.3 ($CH_{(\gamma, Leu)}$), 22.4 and 21.8 ($CH_{3\,(\delta, Leu)}$); IR (KBr): ν = 3315 and 3270 (NH), 1743 (COOMe), 1650 (amide I), 1536 and 1512 (amide II) cm^{-1}; $C_{32}H_{42}N_4O_8$ (610.668): calcd. C 62.93, H 6.93, N 9.18; found C 63.00, H 6.97, N 9.32.

(*S,S*)-*N,N′*-Oxalyl-*bis*(phenylglycyl-phenylglycine methyl ester) (3b); *Following the general procedure the title compound was obtained starting from* H-L-Phg-L-PhgOMe, yield: 66 %; M.p. 272–276 °C (from MeOH–CH_2Cl_2–ether); $[\alpha]_D^{20}$ = +154 (*c* 0.5 in MeOH–CH_2Cl_2, 2:3); ^{1}H NMR (300 MHz, [D_6]DMSO, 20 °C): δ = 9.35 (d, $^3J_{H,H}$ = 6.7 Hz, 2 H, NH), 8.76 (d, $^3J_{H,H}$ = 8.5 Hz, 2 H, NH), 7.47–7.30

(m, 20 H, H_{arom}), 6.71 (d, $^3J_{H,H}$ = 8.5 Hz, 2 H, CH_α), 5.47 (d, $^3J_{H,H}$ = 6.7 Hz, 2 H, CH_α), 3.57 (s, 6 H, $CH_{3\ (OMe)}$); ^{13}C NMR (75.5 MHz, [D$_6$]DMSO), 20 °C): δ = 170.9 and 169.0 (CONH and COOMe), 158.8 ($CONH_{oxal}$), 137.6, 136.0, 129.1, 128.8, 128.3, 128.1 and 127.5 (C_{arom}), 56.7 and 55.8 (CH_α), 52.4 ($CH_{3\ (OMe)}$); IR (KBr): ν = 3330 br and 3280 br (NH), 1744 (COOMe), 1650 (amide I), 1536 and 1497 (amide II) cm^{-1}; $C_{36}H_{34}N_4O_8$ (650.664): calcd. C 66.45, H 5.27, N 8.61; found C 66.37, H 5.05, N 8.66.

(*S,S*)-*N,N'*-Oxalyl-*bis*(phenylglycyl-leucine methyl ester) (4b); *Following the general procedure the title compound was obtained starting from* (*S,S*)-Phg-LeuOMe, and the product was purified by the preparative TLC (2.5% MeOH–CH_2Cl_2), yield: 59.3 %; M.p. 218–224 °C (from CH_2Cl_2–light petroleum); $[\alpha]_D^{20}$ = +104 (*c* 0.5 in CH_2Cl_2); ^{1}H NMR (300 MHz, $CDCl_3$, 20 °C, TMS): δ = 8.61 (d, $^3J_{H,H}$ = 7.9 Hz, 2 H, NH_{Phg}), 7.38 - 7.26 (m, l0 H, H_{arom}), 6.46 (d, $^3J_{H,H}$ = 8.1 Hz, 2 H, NH_{Leu}), 5.58 (d, $^3J_{H,H}$ = 7.9 Hz, 2 H, $CH_{\alpha(Phg)}$), 4.62 - 4.58 (m, 2 H, $CH_{\alpha\ (Leu)}$), 3.61 (s, 6 H, $CH_{3\ (OMe)}$), 1.64–1.49 (m, 6 H, $CH_{2\ (\beta,\ Leu)}$ and $CH_{\gamma,\ Leu}$), 0.92 (d, $^3J_{H,H}$ = 4.2 Hz, 12 H, $CH_{3\ (\delta,\ Leu)}$); ^{13}C NMR (75.5 MHz, $CDCl_3$, 20 °C): δ = 173.0 and 168.6 (CONH and COOMe), 158.7 ($CONH_{oxal}$), 136.3, 128.8, 128.5 and 127.5 (C_{arom}), 56.9 ($CH_{\alpha,\ Phg}$), 52.0 ($CH_{3\ (OMe)}$), 50.9 ($CH_{\alpha,\ Leu}$), 41.3 ($CH_{2\ (\beta,\ Leu)}$), 24.5 ($CH_{\gamma,\ Leu}$), 22.4 and 21.7 ($CH_{3\ (\delta,\ Leu)}$); IR (KBr): ν = 3320 and 3274 (NH), 1744 (COOH), 1658 (amide I), 1550 and 1498 (amide II) cm^{-1}; $C_{32}H_{42}N_4O_8{\cdot}2H_2O$ (646.72): C 59.43, H 7.17, N 8.66; found C 59.25, H 6.88, N 8.77.

(*S,S*)-*N,N'*-Oxalyl-*bis*(phenylalanyl-phenylalanine methyl ester) (5b); *Following the general procedure the title compound was obtained starting from* **(*S,S*)**-Phe-PheOMe, yield: 67 %; M.p. 241 - 243 °C (from acetonitrile); $[\alpha]_D^{20}$ = + 154 (*c* 0.5 in MeOH - CH_2Cl_2, 2:3); ^{1}H NMR (300 MHz, [D$_6$]DMSO, 20 °C): δ = 8.64 (d, $^3J_{H,H}$ = 7.8 Hz, 2 H; NH), 8.38 (d, $^3J_{H,H}$ = 8.9 Hz, 2 H; NH), 7.28 - 7.07 (m, 20 H, H_{arom}), 4.54-4.48 (m, 4 H; CH_α), 3.60 (s, 6 H; $CH_{3(OMe)}$), 3.06 (dd, , $^3J_{H,H}$ = 13.8 and $^2J_{H,H}$ =

5.5 Hz, 2H, $CH_{2(\beta)}$), and 2.97-2.87 (m, 6H, $CH_{2(\beta)}$); ^{13}C NMR (75.5 MHz, [D_6]DMSO), 20 °C): δ = 171.6 and 169.9 (CONH and COOMe), 158.6 ($CONH_{oxal}$), 137.0, 136.9, 129.1, 129.0, 128.2, 128.0 , 126.6 and 1263 (C_{arom}), 53.9 and 53.5 (CH_{α}), 51.9 ($CH_{3(OMe)}$), 37.1 and 36.7 $CH_{2(\beta)}$); IR (KBr): ν = 3330 br and 3280 br (NH), 1744 (COOMe), 1650 (amide I), 1536 and 1497 (amide II) cm^{-1}; $C_{40}H_{42}N_40_8$ (706.768): calcd. C 67.97, H 5.99, N 7.93; found C 67.73, H 5.85, N 8.06.

***General procedure for the preparation of oxalamide - dicarboxylic acids* 1a–5a**

A solution of diester **1a–5a** (1 mmol) in 1 M LiOH (3 mL), MeOH (7 mL) and CH_2Cl_2 (2 mL) was stirred for 17 h at room temperature. The reaction mixture was adjusted to pH 7 with 1 M HC1 and the most of the solvent evaporated *in vacuo*. Water (4 mL) was added and the mixture acidified (pH 2.5) with 1 M HCl. The precipitate was filtered off, washed with H_2O and dried *in vacuo.*

(*S,S*)-*N,N'*-Oxalyl-*bis*(leucyl-leucine) (1a); *Following the general procedure the title compound was obtained starting from* **1b, y**ield of (*S,S*)-**1a**: 89 %, M.p. 258–260 °C (from MeOH–light petroleum); $[\alpha]_D^{20} = -52$ (*c* 0.5 in MeOH); ^1H NMR (300 MHz, [D_6]DMSO, 20 °C): δ = 8.52 (d, $^3J_{H,H}$ = 8.8 Hz, 2H, NH), 8.28 (d, $^3J_{H,H}$ = 7.6 Hz, 2 H, NH), 4.43–4.35 and 4.25–4.17 (2 m, 2 H each, CH_{α}), 1.66–1.50 (m, 12 H, $CH_{2\,(\beta)}$ and CH_{γ}), 0.89–0.82 (m, 24 H, $CH_{3\,(\delta)}$); ^{13}C NMR (75.5 MHz, CD_3OD), 20 °C): δ = 176.2 and 174.4 (CONH and COOH), 161.4 ($CONH_{oxal}$), 53.5 and 52.3 (CH_{α}), 42.4 and 41.8 ($CH_{2\,(\beta)}$), 26.17 and 26.11 (CH_{γ}), 23.65, 23.61, 22.4 and 22.1 ($CH_{3\,(\delta)}$); IR (KBr): ν = 3275 (NH), 1724 (COOH), 1659 (amide I), 1539 and 1512 (amide II) cm^{-1}; $C_{26}H_{46}N_4O_8 \cdot H_2O$ (560.676): calcd C 55.69, H 8.63, N 9.99; found C 55.78, H 8.59, N 9.96.

(*S,R*)-*N,N'*-Oxalyl-*bis*(leucyl-leucine) 1a and **(*R,S*)-*N,N'*-Oxalyl-*bis*(leucyl-leucine) 1a** were prepared following the same procedure, starting from (*S,R*)-*N,N'*-Oxalyl-bis(leucyl-leucine methyl ester) (**1b)** and (*R,S*)-*N,N'*-Oxalyl-bis(leucyl-leucine methyl ester) (**1b**), respectively.

(*S,S*)-*N,N'*-Oxalyl-*bis*-(leucyl-phenylglycine) (2a); *Following the general procedure the title compound was obtained starting from* **2b, y**ield: 96 %; M.p. 220–227 °C (xerogel from MeOH–CH_2Cl_2–light petroleum); $[\alpha]_D^{20} = -1$ (*c* 1 in MeOH); ^{1}H NMR (300 MHz, [D$_6$]DMSO, 20 °C): δ = 8.87–8.79 and 8.69–8.60 (2 m, 2 H each, NH), 7.38 (s, 10 H, H_{arom}), 5.29 (d, $^3J_{H,H}$ = 5.5 Hz, 2 H, $CH_{\alpha, Phg}$), 4.56–4.44 (m, 2 H, $CH_{\alpha, Leu}$), 1.74–1.41 (m, 6H, $CH_{2\ (\beta, Leu)}$ and $CH_{\gamma, Leu}$), 0.89–0.84 (m, 12 H, $CH_{3\ (\delta)}$); ^{13}C NMR (75.5 MHz, CD_3OD, 20 °C): δ = 173.9 (CONH and COOH), 161.5 ($CONH_{oxal}$), 138.2, 130.2, 129.8 and 129.2 (C_{arom}), 58.4 ($CH_{\alpha, Phg}$), 53.5 ($CH_{\alpha, Leu}$), 42.4 ($CH_{2\ (\beta, Leu)}$), 26.1 ($CH_{\gamma, Leu}$), 23.6 and 22.5 ($CH_{3\ (\delta, Leu)}$); IR (KBr): ν = 3320 br (NH), 1735 (COOH), 1660 (amide I), 1505 (amide II) cm^{-1}; $C_{3o}H_{38}N_4O_6$ (582.636): C 61.84, H 6.57, N 9.62: found; C 61.96, H 6.42, N 9.74.

(*S,S*)-*N,N'*-Oxalyl-*bis*(phenylglycyl-phenylglycine) (3a); *Following the general procedure the title compound was obtained starting from* **3b, y**ield: 79 %; M.p. 252–254 °C (from MeOH–CH_2Cl_2–light petroleum); $[\alpha]_D^{20} = 0$ (*c* 0.4 in MeOH:CH_2Cl_2 2:3); ^{1}H NMR (300 MHz, [D$_6$]DMSO, 20 °C): δ = 9.28–9.24 and 8.80–8.73 (2 m, 2 H each, NH), 7.47–7.28 (m, 20 H, H_{arom}), 5.78–5.69 and 5.38–5.32 (2 m, 2 H each, CH_α); ^{13}C NMR (75.5 MHz, [D$_6$]DMSO), 20 °C): δ = 171.8, 171.7 and 169.1 (CONH and COOH), 158.8 ($CONH_{oxal}$), 138.0, 137.1, 136.8, 128.9, 128.8, 128.4, 128.3, 128.0, 127.7, 127.5 and 127.3 (C_{arom}), 56.7 and 55.9 (CH_α); IR (KBr): ν = 3300 br (NH), 1740 br (COOH), 1660 br (amide I), 1500 (amide II) cm^{-1}; $C_{34}H_{3o}N_4O_8$ (622.612): calcd C 65.58, H 4.86, N 9.00; found C 65.42, H 4.60, N 9.01.

(*S,S*)-*N,N'*-Oxalyl-*bis*(phenylglycyl-leucine) (4a); *Following the general procedure the title compound was obtained starting from* **4b, y**ield: 65 %; M.p. 253–257 °C (xerogel from MeOH–CH_2Cl_2); $[\alpha]_D^{20}$ = −62 (*c* 0.5 in MeOH–CH_2Cl_2 3:2); ^{1}H NMR (300 MHz, [D$_6$]DMSO, 20 °C): δ = 8.80–8.64 (m, 4 H, NH), 7.42–7.30 (m, 10 H, H_{arom}), 5.60–5.53 (m, 2 H, $CH_{\alpha,\ Phg}$), 4.31–4.12 (m, 2 H, $CH_{\alpha,\ Leu}$), 1.58–1.24 (m, 6H, $CH_{2\ (\beta,\ Leu)}$ and $CH_{\gamma,\ Leu}$), 0.96–0.61 (m, 12 H; $CH_{3\ (\delta,\ Leu)}$); ^{13}C NMR (75.5 MHz, CD_3OD, 20 °C): δ = 175.9, 176.2 and 171.9 (CONH and COOH), 160.7 ($CONH_{oxal}$), 139.1, 138.4, 130.2, 129.9, 129.0, 128.9 and 128.86 (C_{arom}), 58.6 and 58.5 ($CH_{\alpha,\ Phg}$), 52.54 and 52.48 ($CH_{\alpha,\ Leu}$), 41.8 and 41.6 ($CH_{2\ (\beta,\ Leu)}$), 26.2 and 26.1 ($CH_{\gamma,\ Leu}$), 23.6, 22.1 and 21.6 ($CH_{3\ (\delta,\ Leu)}$); IR (KBr): ν = 3317 and 3278 (NH), 1745, (COOH), 1658 (amide I), 1548 and 1500 (amide II) cm^{-1}; $C_{3o}H_{38}N_4O_8$ (582.636): C 61.84, H 6.57, N 9.62; found C 61.79, H 6.73, N 9.78.

(*S,S*)-*N,N'*-Oxalyl-*bis*(phenylalanyl-phenylalanine) (5a); *Following the general procedure the title compound was obtained starting from* **5b,** yield:82 %; M.p:257-259°C (from MeOH-CH_2Cl_2-light petroleum); $[\alpha]_D^{20}$ =+8 (*c* 0.5, DMSO);^{1}H NMR (300 MHz, [D$_6$]DMSO, 20 °C): δ = 12.86 (2H, br s, OH), 8.50 (d, $^3J_{H,H}$ = 7.98 Hz, 2 H; NH), 8.36 (d, $^3J_{H,H}$ = 8.98 Hz, 2 H; NH), 7.30 - 7.06 (m, 20 H, H_{arom}), 4.55-4.42 (m, 4 H; CH_α), 3.08 (dd, ^3J(H,H) = 14.22 and ^2J(H,H) = 4.98 Hz, 2H, $CH_{2(\beta)}$), and 2.98-2.87 (m, 6H, $CH_{2(\beta)}$);^{13}C NMR (75.5 MHz, [D$_6$]DMSO), 20 °C): δ = 172.6 and 169.8 (CONH and COOH), 158.6 ($CONH_{oxal}$), 137.2, 137.0, 129.1, 129.08, 128.2, 128.0 , 126.5 and 126.3 (C_{arom}), 53.9 and 53.4 (CH_α), 37.1 and 36.7 $CH_{2(\beta)}$); IR (KBr): ν = 3381 br and 3269 br (NH), 1719 (COOH), 1649 (amide I), 1540, 1505 and 1497 (amide II) cm^{-1}; $C_{38}H_{38}N_40_8$ (678.716): calcd C 67.24, H 5.64, N 8.26; found C 67.13, H 5.81, N 8.24.

***General procedure for the preparation of oxalamide - diamides* 1c – 5c**

A solution of diester **1b–5b** (1 mmol) in conc. NH_3/MeOH (50 mL) was kept for 7 days at 7 ^{o}C. The precipitate was filtered off and washed with MeOH.

(*S,S*)-*N,N'*-Oxalyl-*bis*(leucyl-leucinamide) (1c); *Following the general procedure the title compound was obtained starting from* **3b, y**ield: 82 %, M.p. 306–308 ^{o}C (from MeOH); $[\alpha]_D^{20}$ = –55 (*c* 0.18 in MeOH); ^{1}H NMR (300 MHz, [D$_6$]DMSO, 20 ^{o}C): δ = 8.59 (d, $^3J_{H,H}$ = 8.5 Hz, 2 H, NH), 8.07 (d, $^3J_{H,H}$ = 8.2 Hz, 2 H, NH), 7.34 and 6.95 (2s, 2 H each, $CONH_2$), 4.42–4.33 and 4.29–4.21, (2 m, 2 H each, CH_α), 1.65–1.41 (m, 12 H, $CH_{2\ (\beta)}$ and CH_γ), 0.89–0.82 (m, 24 H, $CH_{3\ (\delta)}$); ^{13}C NMR (75.5 MHz, [D$_6$]DMSO), 20 ^{o}C): δ = 174.1 and 170.9 (CONH and $CONH_2$), 159.4 ($CONH_{oxal}$), 51.9 and 50.9 (CH_α), 41.1 and 41.0 ($CH_{2\ (\beta)}$), 24.4 and 24.3 (CH_γ), 23.0, 21.70 and 21.65 ($CH_{3\ (\delta)}$); IR (KBr): ν = 3382, 3290, 3263 and 3200 (NH), 1650 br (amide I), 1535 and 1509 (amide II) cm^{-1}; $C_{26}H_{48}N_6O_6 \cdot 2H_2O$ (576.724): calcd C 54.14, H 9.09, N 14.57; found C 53.97, H 8.88, N 14.38.

(*S,S*)-*N,N'*-Oxalyl-*bis*(leucyl-phenylglycinamide) (2c); *Following the general procedure the title compound was obtained starting from* **2b, y**ield: 81 %, M.p. 290–294 ^{o}C (xerogel from DMF–MeOH–CH_2Cl_2–light petroleum); $[\alpha]_D^{20}$ = −45 (*c* 0.7 in DMSO); ^{1}H NMR (300 MHz, [D$_6$]DMSO, 20 ^{o}C): δ = 8.92–8.65 (m, 4 H, NH), 7.76 and 7.21 (2 br s, 2 H each, $CONH_2$), 7.46–7.27 (m, 10 H, H_{arom}), 5.41 and 5.42–5.38 (m, 2 H, $CH_{\alpha,\ Phg}$), 4.58–4.46 (m, 2 H, $CH_{\alpha,\ Leu}$), 1.68–1.42 (m, 6 H, $CH_{2\ (\beta,\ Leu)}$ and $CH_{\gamma,\ Leu}$), 0.85 (d, $^3J_{H,H}$ = 5.2 Hz, 12 H, $CH_{3\ (\delta.\ Leu)}$); ^{13}C NMR (75.5 MHz, [D$_6$]DMSO), 20 ^{o}C): δ = 171.8, 171.1 and 170.9 (CONH and $CONH_2$), 159.6 ($CONH_{oxal}$), 139.2, 128.6, 127.8 and 127.2 (C_{arom}), 56.2 ($CH_{\alpha,\ Phg}$), 52.1 and 52.0 ($CH_{\alpha,\ Leu}$), 40.8 ($CH_{2\ (\beta,\ Leu)}$), 24.5 ($CH_{\gamma,\ Leu)}$), 23.1 and 21.6 ($CH_{3\ (\delta,\ Leu)}$); IR (KBr): ν

= 3310, 3260 and 3218 (NH), 1678, 1659 and 1641 (amide I), 1536, 1512 and 1500 (amide II) cm^{-1}; $C_{30}H_{40}N_6O_6$ (580.668): calcd. C 62.05, H 6.94, N 14.47; found C 62.31, H 6.77, N 14.67.

(*S,S*)-*N,N'*-Oxalyl-*bis*(phenylglycyl-phenylglycinamide) (3c); *Following the general procedure the title compound was obtained starting from* **3b, y**ield: 69 %, M.p. > 300 °C (from DMSO–MeOH); $[\alpha]_D^{20}$ = +5 (*c* 0.4 in DMSO); ^1H NMR (300 MHz, [D_6]DMSO, 20 °C): δ = 8.93 and 8.74, (2 br s, 2 H each, NH), 7.63, 7.55, 7.10 and 7.03, (4 br s, 4 H, $CONH_2$), 7.45 - 7.24 (m, 20 H, H_{arom}), 5.72 (br s, 2 H; CH_α), 5.53–5.37 (m, 2 H, CH_α); ^{13}C NMR (75.5 MHz, [D_6]DMSO, 20 °C): δ = 170.8 and 168.0 (CONH and $CONH_2$), 158.4 ($CONH_{oxal}$), 138.5, 137.7, 128.3, 128.1, 127.8, 127.4, 126.9 and 126.7 (C_{arom}), 56.2 and 55.9 (CH_α); IR (KBr): ν = 3465, 3310 br, 3268 and 3210 br (NH), 1650 br (amide I), 1538, 1513 and 1498 (amide II) cm^{-1}; $C_{34}H_{32}N_6O_6 \cdot 2\ H_2O$ (656.676): calcd C 62.18, H 5.53, N 12.80; found C 62.27, H 5.67, N 12.83.

(*S,S*)-*N,N'*-Oxalyl-*bis*(phenylglycyl-leucinamide) (4c); *Following the general procedure the title compound was obtained starting from* **4b, y**ield: 78 %, M.p. 250–253 °C (xerogel from DMF–CH_2Cl_2–light petroleum); $[\alpha]_D^{20}$ = −16.5 (*c* 0.48 in DMSO); ^1H NMR (300 MHz, [D_6]DMSO, 20 °C): δ = 8.77–8.53 (m, 4 H, NH), 7.50–7.30 (m, 10 H, H_{arom}), 7.49, 7.45, 7.06 and 6.95 (4 br s, 4 H, $CONH_2$), 5.56–5.51 (m, 2 H; $CH_{\alpha,\ Phg}$)), 4.34–4.25 and 4.19–4.10, (2 m, 2 H, $CH_{\alpha,\ Leu}$)), 1.62–1.19 (m, 6 H, $CH_{2\ (\beta,\ Leu)}$ and $CH_{\gamma\ (Leu)}$), 0.89–0.58 (m, 12 H, $CH_{3\ (\delta,\ Leu)}$); ^{13}C NMR (75.5 MHz, [D_6]DMSO), 20 °C): δ = 174.1, 173.6, 168.5 and 167.0 (CONH and $CONH_2$), 158.7 ($CONH_{oxal}$), 138.3, 138.0, 128.7, 128.6, 128.1, 127.2 and 127.1 (C_{arom}), 56.19 and 56.11 ($CH_{\alpha,\ Phg}$), 51.2 ($CH_{\alpha,\ Leu}$), 41.3 and 40.7 ($CH_{2(\beta,\ Leu)}$)), 24.24 and 24.18, 23.0, 21.8 and 21.1 ($CH_{\gamma,\ Leu}$ and $CH_{3\ (\delta,\ Leu)}$); IR (KBr): ν = 3400 br, 3300 br and 3210 br

(NH), 1652 br (amide I), 1540 and 1497 (amide II) cm^{-1}; $C_{30}H_{40}N_6O_6$ (580.668): calcd C 62.05, H 6.94, N 14.47; found C 61.97, H 7.20, N 14.44.

(*S,S*)-*N,N'*-Oxalyl-*bis*(phenylalanyl-phenylalaninamide) (5c); *Following the general procedure the title compound was obtained starting from* 5**b, y**ield: 72%, M.p**.** > 300 °C (from DMSO - MeOH); ^{1}H NMR (300 MHz, [D$_6$]DMSO, 20 °C): δ = 8.37 (d, ^3J(H,H) = 8.9 Hz, 2 H; NH), 8.32 (d, ^3J(H,H) = 8.6 Hz, 2 H; NH), 7.45 (br s, 2 H, $CONH_2$), 7.28-7.00 (m, 22 H; H_{arom} and $CONH_2$), 4.57-4.41 (m, 4 H CH_α), 3.06-2.74 (m, 8H, $CH_{2(\beta)}$); ^{13}C NMR (75.5 MHz, [D$_6$]DMSO, 20 °C): δ == 173.0 and 169.9 (CONH and $CONH_2$), 159.0 ($CONH_{oxal}$), 138.1, 137.5, 129.7, 129.6, 128.49, 128.46 , 126.78 and 126.74 (C_{arom}), 54.6 and 54.3 (CH_α), 38.3 and 37.6 $CH_{2(\beta)}$); IR (KBr): ν = 3384 br and 3268 br (NH), 1675 (COOH), 1649 (amide I), 1530, 1507 and 1453 (amide II) cm^{-1}; HRMS calcd. for $C_{38}H_{40}N_6O_6$ $[M+Na]^+$ 699.2901, found .699.2880.

Gelation experiments: The experiments were performed by dissolution of a weighed amount of the compound in a measured volume of the selected pure solvent or solubilising component. In the case of solvent mixtures, a measured volume of second solvent of lower polarity was added until the gel was formed the procedure being reminiscent of two-solvent crystallization. All gelator effectiveness values (G_{eff}; see also V. Caplar, L. Frkanec, N. Sijakovic Vujicic, M. Zinic, *Chem Eur. J.* 2010, 16, 3066–3082) were determined using 10 mg of tested gelator and test tubes of 10 mm internal diameter (see Chapter 8 of ref. 17). Measured small volumes (µL) of tested solvent were added followed by heating and cooling of the sample after each solvent addition until a loose gel or fluid sample was obtained.

TEM and SEM investigations: transmission electron micrographs were taken on an EM 10 A Zeiss or FEI Morgagni 268D transmission electron microscope. A small amount of sample was placed on carbon-coated grid (copper, 100 mesh). The sample was negatively stained with PWK (dipotassium phosphotungstate).

TEM images of 4a water/DMSO and 5a EtOH gels.

a) b)

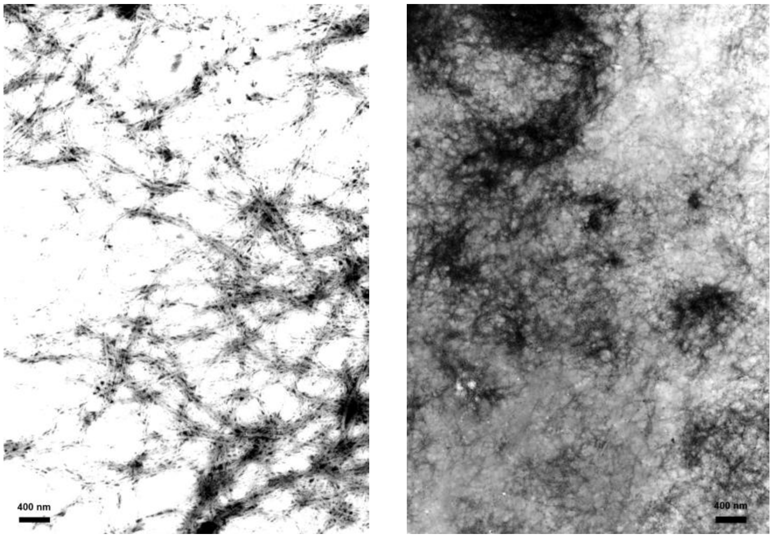

Figure S1: TEM images of; *a)* **4a** gel/(DMSO – H_2O); *b)* **5a** gel (EtOH).

Molecular modeling

a)

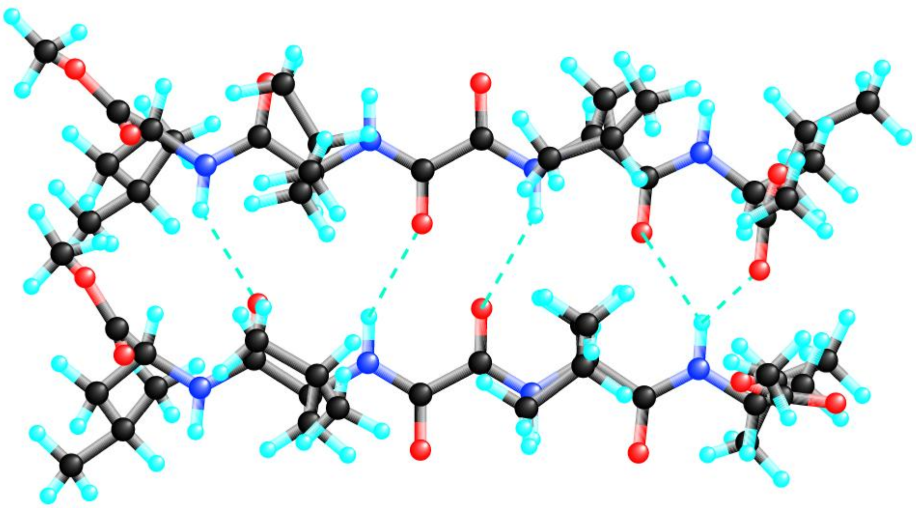

b)

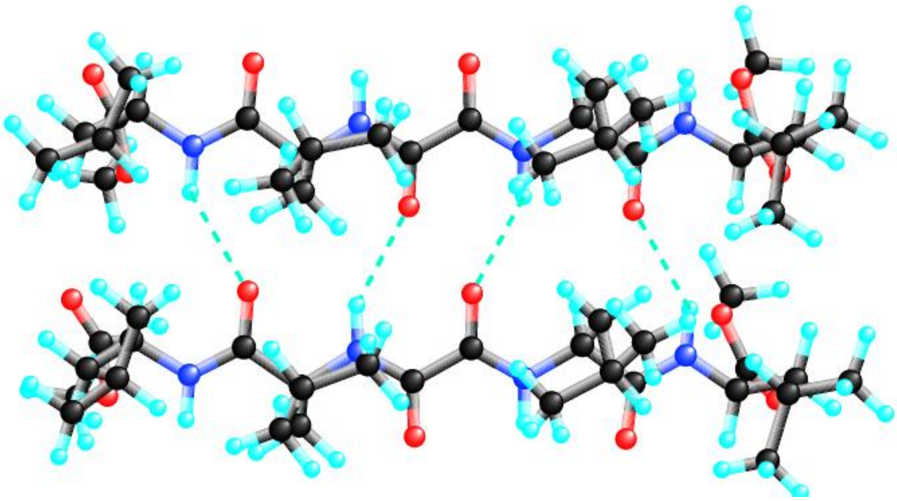

c)

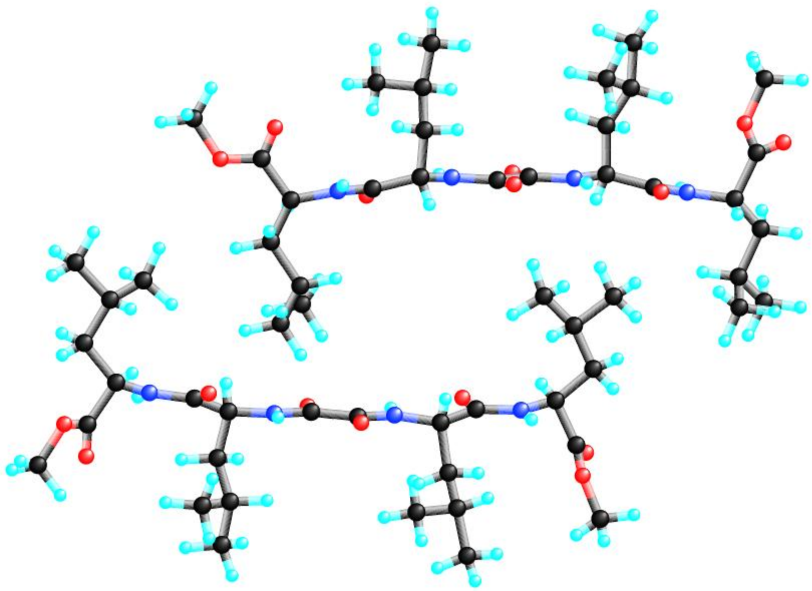

d)

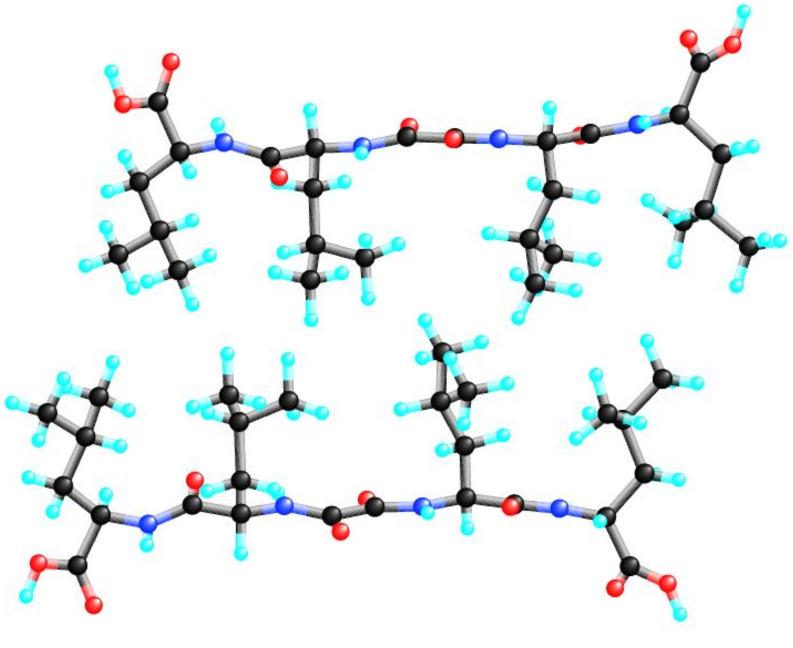

Figure S2: Hydrogen bonded dimers of (a) (*S,S*)-**1b**, (b) (*S,R*)-**1b** and **the** dimers formed by lipophilic interactions (c) (*S,S*)-**1b,** (d) (*S,R*)-**1a** generated by Docking calculations (SYBYL® package of TRIPOS Inc).
